# Longitudinal single-cell RNA-seq analysis reveals stress-promoted chemoresistance in metastatic ovarian cancer

**DOI:** 10.1126/sciadv.abm1831

**Published:** 2022-02-23

**Authors:** Kaiyang Zhang, Erdogan Pekcan Erkan, Sanaz Jamalzadeh, Jun Dai, Noora Andersson, Katja Kaipio, Tarja Lamminen, Naziha Mansuri, Kaisa Huhtinen, Olli Carpén, Sakari Hietanen, Jaana Oikkonen, Johanna Hynninen, Anni Virtanen, Antti Häkkinen, Sampsa Hautaniemi, Anna Vähärautio

**Affiliations:** 1 Research Program in Systems Oncology, Research Programs Unit, Faculty of Medicine, University of Helsinki, Helsinki, Finland.; 2 Cancer Research Unit, Institute of Biomedicine and FICAN West Cancer Centre, University of Turku, Turku, Finland.; 3 Department of Pathology, University of Helsinki and HUSLAB, Helsinki University Hospital, Helsinki, Finland.; 4 Department of Obstetrics and Gynecology, University of Turku and Turku University Hospital, Turku, Finland.; 5 Finnish Cancer Registry, Helsinki, Finland.; 6 Department of Pathology, University of Helsinki and HUS Diagnostic Center, Helsinki University Hospital, Helsinki, Finland.

## Abstract

Chemotherapy resistance is a critical contributor to cancer mortality and thus an urgent unmet challenge in oncology. To characterize chemotherapy resistance processes in high-grade serous ovarian cancer, we prospectively collected tissue samples before and after chemotherapy and analyzed their transcriptomic profiles at a single-cell resolution. After removing patient-specific signals by a novel analysis approach, PRIMUS, we found a consistent increase in stress-associated cell state during chemotherapy, which was validated by RNA in situ hybridization and bulk RNA sequencing. The stress-associated state exists before chemotherapy, is subclonally enriched during the treatment, and associates with poor progression-free survival. Co-occurrence with an inflammatory cancer–associated fibroblast subtype in tumors implies that chemotherapy is associated with stress response in both cancer cells and stroma, driving a paracrine feed-forward loop. In summary, we have found a resistant state that integrates stromal signaling and subclonal evolution and offers targets to overcome chemotherapy resistance.

## Teaser

Ovarian cancer chemotherapy enriches a stressed state associated with poor response on both patient and subclonal levels.

## INTRODUCTION

Platinum-based chemotherapy is the most widely prescribed drug in metastatic cancer treatment (*1*). It is curative in testicular cancers and effective in other cancers, such as in high-grade serous ovarian cancer (HGSOC) where the introduction of platinum-based combination therapy improved the 10-year survival rate by more than 10% and doubled the number of complete responses (*1*, *2*). However, most patients with HGSOC develop platinum resistance leading to almost invariably fatal refractory disease and only 43% 5-year survival (*3*). HGSOC is a copy number–driven cancer that has exceptionally high intratumor heterogeneity and almost 100% prevalence of *TP53* mutations (*4*, *5*), which impedes overcoming platinum resistance.

Patients with platinum-sensitive HGSOC with homologous recombination–deficient (HRD) tumors benefit from poly(adenosine diphosphate–ribose) polymerase (PARP) inhibitors (*6*). However, approximately half of the patients with HGSOC do not have HRD tumors and face very limited treatment options at the chemotherapy-resistant stage. On cellular level, clinically observed chemotherapy resistance is a continuum from a Darwinian selection process of intrinsically resistant cell populations to an adaptive induction of a fitness phenotype (*7*, *8*). Most studies of drug resistance in the clinical setting have so far focused on genetic changes, such as *MET* amplification with kinase inhibitors (*9*), *BRCA* reversal mutations with chemotherapy (*10*), or genomic signatures in a heterogeneously treated patient cohort (*11*). The number and complexity of resistance mechanisms to chemotherapy surpass those of targeted therapies (*12*), which warrant homogeneously treated patient cohorts that allow high-resolution analysis of cancer cells before and after chemotherapy.

Chemotherapy affects transcriptional programs of cancer cells, which provides an opportunity to comprehensively decipher the most relevant chemotherapy-induced processes using single-cell RNA sequencing (scRNA-seq) data. Data from scRNA-seq also enable addressing the interplay between cancer cells and tumor microenvironment (TME). scRNA-seq and genomic analysis performed before and after treatment in paired samples from four patients with metastatic breast cancer revealed that while chemotherapy selected preexisting genetic abnormalities, it also induced adaptive transcriptional changes related to epithelial-to-mesenchymal transition (EMT), *AKT1* signaling, and hypoxia (*13*). In paired samples from four patients with non–small cell lung cancer, the surviving cells underwent a primitive state change to alveolar cells in residual disease (*14*). While these studies demonstrate the importance of paired samples, they each had cancer cells containing pairwise specimens from only four patients and, more importantly, limited clinical data from the patients, such as the patient outcome after therapy or survival times, which hinders making clinically relevant conclusions from the data.

Here, we characterized transcriptional patterns of chemotherapy resistance in HGSOC using patient-derived prospective tissue sample pairs before and after treatment at single-cell resolution. Our cohort consists of scRNA-seq data from treatment-naïve and post–neoadjuvant chemotherapy (post-NACT) pairs from 11 homogeneously treated patients with HGSOC with full clinical information. To validate our findings, we used RNA in situ hybridization (RNA-ISH) data of 10 treatment-naïve versus post-NACT sample pairs, 49 bulk RNA-seq samples including 18 treatment-naïve versus post-NACT pairs, and 8 treatment-naïve versus relapse pairs in the HERCULES cohort (http://project-hercules.eu/) and bulk RNA-seq data of 271 treatment-naïve samples in The Cancer Genome Atlas (TCGA) cohort (*5*). Our unbiased analysis reveals how chemotherapy modulates cancer cell states by both subclonal selection and microenvironment-boosted transcriptional induction across the homogeneously treated sample cohort. Our results define a cell state that allows biomarker-based prediction and targeting of chemoresistance.

## RESULTS

### Obtaining scRNA-seq data from HGSOC patient samples before and after chemotherapy

**Fig. 1.**
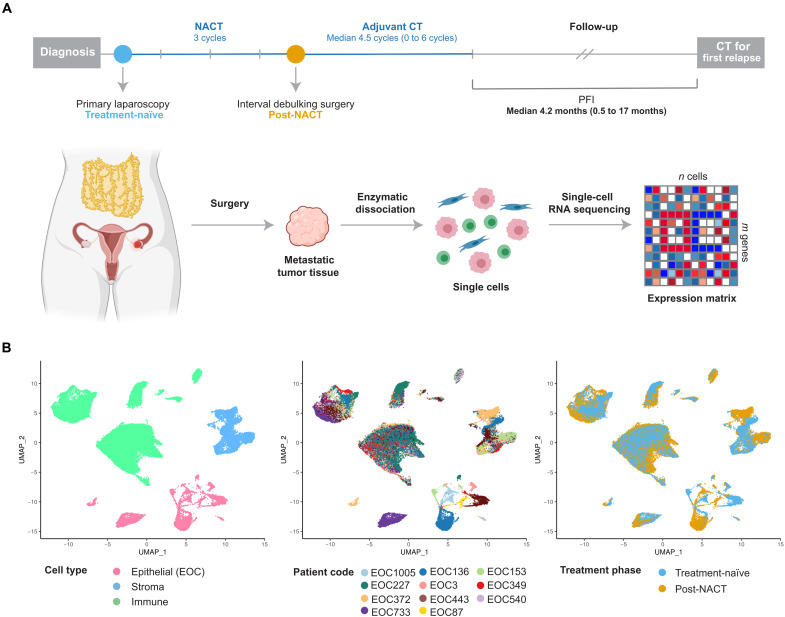
Overview of experimental and sequencing workflow. (**A**) Diagram showing the sample collection and processing. We collected prospective tumor samples from 11 patients with HGSOC before and after NACT. The median PFI in the cohort was 4.2 months. scRNA-seq was performed on dissociated solid tumor specimens using the 10x Genomics Chromium platform. (**B**) Uniform manifold approximation and projection (UMAP) plot of all cells (*n* = 51,786) passing the quality control, colored by cell type, patient code, and treatment phase. EOC, epithelial ovarian carcinoma.

We collected prospective tissue samples from 11 patients with HGSOC before and after chemotherapy and measured transcriptomes of 93,650 cells using scRNA-seq (Fig. 1A and see Materials and Methods). All patients in the study were treated with NACT, i.e., diagnostic laparoscopy followed by three cycles of platinum-taxane, interval debulking surgery (IDS), and adjuvant chemotherapy, and four patients further received bevacizumab maintenance therapy. NACT is typically recommended for patients who are inoperable at diagnosis and often have poor prognosis. Accordingly, in our cohort, the median platinum-free interval (PFI; Fig. 1A), which measures the time from treatment end to relapse, is only 4.2 months. Our sample cohort with metastatic tumors from poorly responsive patients represents many understudied aspects of HGSOC as described in Materials and Methods. Further clinical information of the cohort is given in Table 1.

**Table 1.** Patient and sample information. PDS, primary debulking surgery; NA, not available; CRS, chemotherapy response score; TN, treatment-naïve; PN, post-NACT.

**Patient ID**	Age*	**Treatment**	**Stage^†^**	**PFI (days)**	**CRS**	**CA125 (U/ml)**		**Anatomical locations^‡^**				
						**TN**	**PN**	**scRNA-seq TN**	**scRNA-seq PN**	**Bulk RNA-seq TN**	**Bulk RNA-seq PN**	**Bulk RNA-seq relapse**
**EOC1005**	73	NACT	IVA	65	2	3776	343	Peritoneum	Tumor^§^	NA	NA	NA
**EOC136**	64	NACT	IVA	520	2	2647	212	Mesentery	Omentum	NA	NA	NA
**EOC153**	78	NACT	IVA	393	2	1063	93	Omentum	Omentum	NA	NA	NA
**EOC227**	74	NACT	IVA	230	2	445	33	Omentum^§^	Omentum^§^	NA	NA	NA
**EOC3**	67	NACT	IVA	14	2	821	221	Peritoneum^§^	Omentum	Peritoneum	Omentum	NA
**EOC349**	67	NACT	IVB	36	2	2155	67	Peritoneum^§^	Omentum^§^	NA	NA	NA
**EOC372**	68	NACT	IIIC	460	1	3180	334	Peritoneum	Peritoneum	Peritoneum	Peritoneum	NA
**EOC443**	54	NACT	IVA	177	3	2295	82	Omentum	Omentum	Omentum	Omentum	NA
**EOC540**	62	NACT	IIIC	126	2	155	7	Omentum	Omentum	NA	NA	NA
**EOC733**	72	NACT	IVA	83	1	22079	3579	Peritoneum	Omentum	NA	NA	NA
**EOC87**	62	NACT	IIIC	30	1	998	346	Peritoneum	Omentum	Omentum	Omentum	NA
**EOC1129**	75	NACT	IIIC	210	NA	3493	212	NA	NA	Omentum	Mesentery	NA
**EOC160**	68	PDS	IVB	648	NA	145	NA	NA	NA	Omentum	NA	Mesentery
**EOC183**	68	NACT	IIIC	203	2	411	15	NA	NA	Omentum	Omentum	NA
**EOC218**	71	NACT	IIIC	974	2	2633	38	NA	NA	Omentum	Omentum	NA
**EOC26**	72	NACT	IIIC	0	2	553	55	NA	NA	Omentum	Omentum	Ascites
**EOC376**	67	NACT	IIIC	280	1	615	20	NA	NA	Omentum	Omentum	NA
**EOC423**	81	NACT	IIIC	721^||^	2	854	20	NA	NA	Omentum	Ovary	NA
**EOC568**	57	NACT	IVA	210	3	192	70	NA	NA	Mesentery	Omentum	NA
**EOC587**	71	NACT	IVB	27	3	149	22	NA	NA	Peritoneum	Tumor	NA
**EOC649**	77	NACT	IVB	511	2	588	22	NA	NA	Peritoneum	Omentum	NA
**EOC677**	68	NACT	IIIC	81	2	1593	11	NA	NA	Peritoneum	Peritoneum	Ascites
**EOC883**	74	NACT	IIIC	91	NA	1515	NA	NA	NA	Adnexa	Ascites	NA
**EOC891**	71	NACT	IIIC	183	NA	687	NA	NA	NA	Omentum	NA	Ascites
**EOC933**	74	NACT	IIIC	632	3	296	13	NA	NA	Peritoneum	Mesentery	NA
**EOC868**	62	NACT	IIIC	285	2	1565	36	NA	NA	Peritoneum	Omentum	Peritoneum
**EOC1133**	66	NACT	IVB	661	2	1156	22	NA	NA	Peritoneum	Omentum	NA
**EOC167**	75	NACT	IIIC	19	NA	5614	NA	NA	NA	Omentum	NA	Ascites
**EOC295**	66	NACT	IIIC	221	2	320	74	NA	NA	Peritoneum	NA	Ascites
**EOC752**	64	NACT	IIIC	174	2	2341	58	NA	NA	Peritoneum	NA	Ascites

*Age at diagnosis, years.

†Tumor staging was performed according to the International Federation of Gynecology and Obstetrics 2014 guidelines.

‡Anatomical locations from which the samples were collected for analyses.

§Cell suspension was stored as frozen before scRNA-seq processing.

||No progression, PFI at outcome update.

After quality control (see Materials and Methods and fig. S1, A to D), we obtained a total of 51,786 cells, including 8806 malignant epithelial (tumor), 8045 stromal, and 34,935 immune cells for the subsequent analyses. We identified epithelial, stromal, and immune cells based on graph-based clustering (*15*) and acknowledged markers (fig. S1B). In contrast to stromal and immune cells, where cells from different patients grouped together, cancer cells exhibited a patient-specific expression pattern (Fig. 1B), similar to previous studies (*14*, *16*, *17*).

### PRIMUS identifies phenotypic groups from heterogenous scRNA-seq datasets

**Fig. 2.**
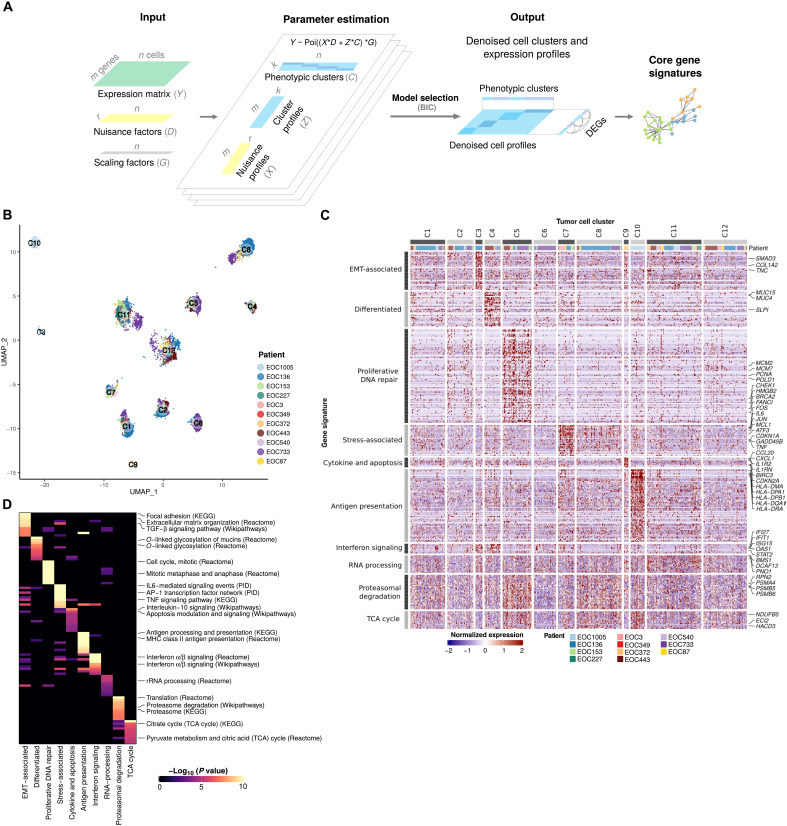
Identification of 12 subpopulations of HGSOC cancer cells characterized by 10 gene signatures. (**A**) Schematic of the PRIMUS model. PRIMUS models the observed single-cell expression profiles (*Y*) as a mixture of latent phenotypic cluster profiles and nuisance profiles. Given *Y*, the known nuisance factors *D*, known size factors *G*, and the number of latent phenotypic clusters *k*, PRIMUS estimates the latent nuisance profiles *X*, latent phenotypic cluster profiles *Z*, and the latent cluster memberships *C* using an expectation-maximization (EM) algorithm. (**B**) UMAP plot of cancer cells after removing the nuisance signals, colored by patient and labeled by the identified clusters. (**C**) Heatmap of the expression of the 10 distinct gene signatures in the 12 identified cell clusters. Rows correspond to genes and columns to cells. (**D**) Heatmap shows the top 10 pathways enriched in each gene signature. TGF-β, transforming growth factor–β; AP1, activating protein 1; TNF, tumor necrosis factor; rRNA, ribosomal RNA; KEGG, Kyoto Encyclopedia of Genes and Genomes; PID, the Pathway Interaction Database.

The observed strong interpatient heterogeneity in cancer cells from genetically divergent cancer samples impedes the direct comparison of transcriptomes across patients. To address this challenge, we developed PRIMUS (Poisson scRNA integration of mixed unknown signals), a holistic clustering approach that identifies phenotypic cell groups from the scRNA-seq data while accounting for patient-specific components and technical noise (Fig. 2A). Specifically, as input, PRIMUS takes scRNA-seq datasets from multiple patients, a design matrix encoding the different nuisance factors, such as patient labels, technical factors (e.g., scRNA-seq quality control metrics), and a vector of size factors. PRIMUS then uses a bilinear Poisson regression model to simultaneously factorize the expression data into the defined nuisance factors, undefined cellular phenotypes, and their corresponding transcriptomic profiles (see Materials and Methods and the Supplementary Materials). As a statistical model, PRIMUS also allows the selection of an optimal number of clusters based on Bayesian information criterion (BIC).

We compared the performance of PRIMUS with existing integration methods (*15*, *18*–*23*) on simulated data and multistudy pancreatic datasets (fig. S2). We simulated datasets containing five cell groups from six samples with different genetic backgrounds and sample-specific effects using splatPop (*24*, *25*) under three scenarios (table S1): (i) All six samples contain the five cell groups; (ii) each sample only contains a subset of cell groups, three pairs of samples had no cell groups in common, and there was one sample-specific cell group; and (iii) the same setting with scenario ii but with unbalanced cell numbers in each sample (from 20 to 2000). For all simulated scenarios and for the pancreatic datasets, PRIMUS was able to accurately cluster cells based on latent cell groups across different samples (fig. S2, A to H). It showed similarly good performance as other existing methods in scenario i, where all samples have the same cell group composition, and performed better than other methods in scenarios ii and iii as well as the real pancreatic datasets, which present sample-specific cell groups/types, and some samples do not have any cell groups/types in common (fig. S2I).

Our results from simulated and pancreatic datasets show that PRIMUS can accurately cluster cells by phenotypic groups, accounting for data source–specific effects from distinct samples. Unlike existing methods, PRIMUS is robust to heterogeneous cell compositions and unbalanced number of cells in different sources, and it also preserves data source–specific cell groups if such are present. Therefore, PRIMUS is a well-justified choice for clustering datasets with potentially unbalanced presentation of phenotypic groups, such as cancer cell states within heterogeneous tumor specimens.

### Identification and characterization of cancer cell states in HGSOC

By using PRIMUS to control the effect of patient-specific variability and technical confounders, such as the percentage of unique molecular identifier (UMI) counts originating from mitochondrial genes, we identified 12 cancer cell clusters (fig. S3A), including three patient-specific clusters (C3, C9, and C10) and nine shared clusters across multiple patients (Fig. 2, B and C). The proportion and number of cells in each cluster from each patient are presented in fig. S3 (B and C, respectively).

To characterize the identified cancer cell clusters, we first identified 4742 significantly differentially expressed genes (DEGs) between at least one pair of the 12 clusters using a likelihood-ratio test (LRT) [false discovery rate (FDR) < 0.01; see Materials and Methods]. To construct well-annotated gene coexpression signatures, we built a gene network using the DEGs integrated to a gene annotation database (*26*) and identified 10 distinct gene signatures after filtering (see Materials and Methods, Fig. 2C, and fig. S3D). Four of 12 clusters (C1, C2, C6, and C12) had no overrepresented gene signatures, suggesting that their DEGs were incoherent, with only limited coexpression and/or poorly annotated, and were thus excluded from further analysis. The remaining eight clusters were characterized by the 10 distinct gene signatures (Fig. 2C and fig. S3E).

Pathway analysis showed that the 10 signatures were associated with diverse biological processes (Fig. 2D). These include key processes previously identified in HGSOC tumors, such as differentiation in cluster C4, proliferation and DNA repair in cluster C5, and EMT identified in the patient-specific cluster C3 (*5*, *27*). We also identified a major histocompatibility complex (MHC) class II antigen presentation signature with high *HLA-DPA1*, *HLA-DQA1*, and *HLA-DRA* expression in the patient-specific cluster C10. Although MHC class II expression is classically considered a feature of professional antigen presenting immune cells, it was recently identified in single HGSOC and normal fallopian tube epithelial cells by Izar *et al.* (*28*) and Hu *et al.* (*27*). Aforementioned studies also identified signatures associated with stress response but excluded them from further analysis as likely artefactual. In our dataset, stress-associated signature, overexpressed by cluster C7, not only consisted of stress-responsive immediate early genes (IEGs) (e.g., *CEBPB*, *FOS*, and *JUN*) but also contained proinflammatory cytokines and receptors [e.g., *IL6*, *TNF*, and *CXCR4*], core transcriptomic regulators of EMT (e.g., *SNAI1* and *SNAI2*), and stemness (*HES1* and *ID2*), as well as prosurvival (e.g., *GADD45B*, *GADD45G*, and *MCL1*) and antiproliferative (*CDKN1A*) genes. Notably, many genes in this signature, such as *IL6*, *TNF*, *CEBPD*, *ATF3*, *NFKBIA*, *BCL6*, *GADD45B*, *GADD45G*, *MCL1*, and *CDKN1A*, are targets of the transcription factor nuclear factor κB (NF-κB). In addition to the cluster-specific signatures described above, we identified three metabolism-associated signatures that were shared by several clusters, representing tricarboxylic acid cycle (TCA), proteasomal degradation, and RNA processing (Table 2).

**Table 2.** Annotation of tumor cell clusters.

**Cell cluster**	**Characteristic gene signature**	**Representative pathways**	**Marker genes**
C3	EMT-associated (43 genes)	TGF-β signaling pathway, focal adhesion	*SMAD3*, *COL1A2*, *TNC*
C4	Differentiated (40 genes)	*O*-linked glycosylation of mucins	*MUC4*, *MUC16*, *SLPI*
C5	Proliferative DNA repair (106 genes)	Cell cycle, DNA repair, Homology directed repair (HDR) through homologous recombination, Fanconi anemia pathway	*PCNA*, *CHEK1*, *HMGB2*, *BRCA2*, *FANCI*, *POLD1*
C7	Stress-associated (35 genes)	IL6-mediated signaling events, TNF signaling pathway, cellular responses to stress	*JUN*, *FOS*, *IL6*, *TNF*, *CXCR4*, *SNAI1*, *VIM*, *GADD45B*, *MCL1*
C9	Cytokine and apoptosis (11 genes)	IL10 signaling, apoptosis modulation and signaling	*CXCL1*, *CCL20*, *IL1R2*, *BIRC3*, *CDKN2A*, *BIK*
C10	Antigen presentation (82 genes)	Antigen processing and presentation, MHC class II antigen presentation	*HLA-DPA1*, *HLA-DQA1*, *HLA-DRA*
C3, C4	Interferon signaling (11 genes)	Interferon signaling	*STAT2*, *IFI27*, *IFIT1*, *OAS1*, *ISG15*
C3, C11	RNA processing (20 genes)	rRNA processing, apoptotic cleavage of cellular proteins	*DCAF13*, *PNO1*, *BMS1*, *ACIN1*, *TJP1*, *ROCK1*
C5, C8	Proteasomal degradation (39 genes)	Proteasome degradation, proteasome complex	*PSMA4*, *PSMB5*, *PSMB6*, *RPN2*
C5, C8, C10	TCA cycle (20 genes)	Citrate cycle (TCA cycle), pyruvate metabolism	*HACD3*, *NDUFB5*, *ECI2*

### Chemotherapy affects the prevalence of proliferative and stress-associated cancer cell populations

**Fig. 3.**
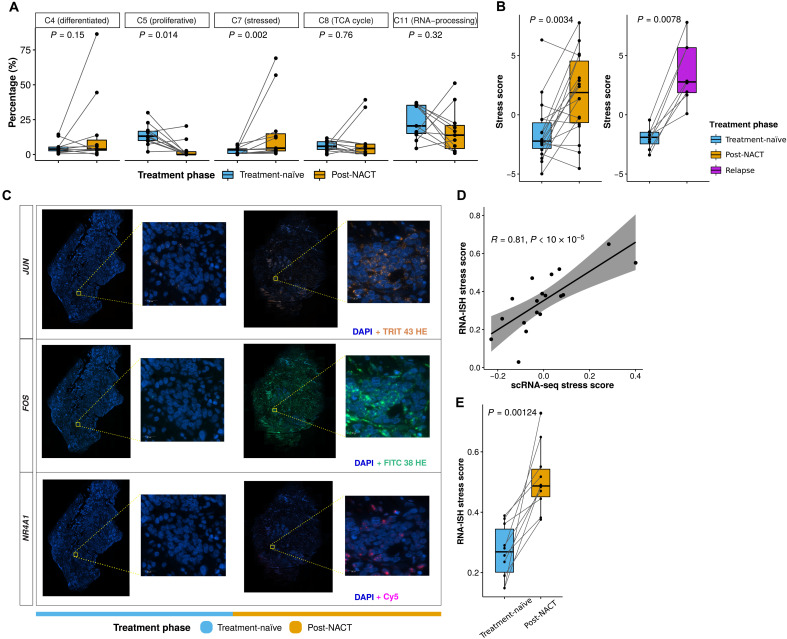
Stress-associated transcriptional profile is enriched after chemotherapy. (**A**) Boxplots showing the fractional changes of the five tumor clusters containing cells from multiple patients, between the treatment-naïve (blue) and post-NACT (yellow) samples of each patient (paired Wilcoxon rank-sum test). Horizontal bars show median values, box edges represent the interquartile range, and each dot represents a sample. (**B**) Boxplots comparing the stress scores in treatment-naïve (blue) versus post-NACT (yellow) samples (left; paired Wilcoxon rank-sum test, *P* = 0.0034), and treatment-naïve (blue) versus relapse (purple) samples (right; paired Wilcoxon rank-sum test, *P* = 0.0078) using bulk RNA-seq data from the HERCULES cohort. Horizontal bars show median values, box edges represent the interquartile range, and each dot represents a sample. (**C**) Representative RNA-ISH images showing the changes of *NR4A1*, *FOS*, and *JUN* from the treatment-naïve to post-NACT sample of patient EOC87. Scale bars, 20 μm. (**D**) Scatter plot showing the correlation (*R* = 0.81, permutation test, *P* < 10 × 10^−5^) between stress scores quantified using RNA-ISH and scRNA-seq experiments. Each dot represents a sample. (**E**) Boxplots comparing the RNA-ISH stress scores in treatment-naïve (blue) versus post-NACT (yellow) samples (permutation test, *P* = 0.00124). Each dot represents a sample.

To test the effect of chemotherapy on the identified 12 cancer cell clusters, we examined the fractional changes of the five clusters that contained cells from multiple patients during chemotherapy. Here, we observed significant differences only in the fractions of the populations expressing proliferative DNA repair signature (C5, *P* = 0.014) and the stress-associated signature (C7, *P* = 0.002) between treatment-naïve and post-NACT samples (Fig. 3A).

The significant decline of C5 cells, from an average of 14% in treatment-naïve samples to an average of 3% in post-NACT samples, implies that chemotherapy either kills most of the proliferative cells or induces cell cycle arrest. An interesting exception to this was patient EOC87 whose fraction of proliferative cells increased from 7 to 11% during chemotherapy. The patient showed no histopathologic response to chemotherapy in omentum and poor prognosis with an overall survival (OS) of only 9 months. This poor prognosis was unexpected since she had a somatic, heterogeneous *BRCA2* frameshift deletion (c.1338delG), which is classified in ClinVar (*29*) as likely pathogenic and thus should be indicative of good response to platinum and PARP inhibitors.

The cluster (C7) represented by stress-associated signature was enriched from an average of 3% in treatment-naïve samples to an average of 17% in post-NACT samples, indicating that this cell state was induced and/or more likely to survive through chemotherapy. We further computed a stress score using stress-associated signature (35 genes) for cancer cell–specific expression deconvoluted from bulk RNA-seq data of 18 treatment-naïve versus post-NACT pairs and 8 treatment-naïve versus relapse pairs. Consistently, post-NACT (*P* = 0.0034) and relapse (*P* = 0.0078) samples showed significantly higher stress scores in comparison to treatment-naïve samples (Fig. 3B). Patient EOC87 with a *BRCA2* frameshift deletion and progressive disease after NACT had the highest stress-associated cluster fraction in the treatment-naïve samples (7%), which may partly explain her poor response to chemotherapy.

### Validation of the stress signature with RNA-ISH

To validate the stress-associated signature with an independent measurement technology, we quantified the expression of 10 stress signature genes in 10 treatment-naïve and post-NACT HGSOC sample pairs with RNA-ISH experiments (see Fig. 3C for representative images). We used canonical correlation analysis (CCA) (see Materials and Methods) to define a stress score that is an aggregate of the RNA-ISH expression levels of the 10 genes to quantify the stress status of each sample.

The RNA-ISH stress score was significantly correlated (*R* = 0.81, permutation test, *P* < 10^−5^) with the scRNA-seq stress score in the matched samples (Fig. 3D). Moreover, the post-NACT samples had significantly higher RNA-ISH stress scores in comparison with the treatment-naïve samples (Fig. 3E; permutation test, *P* = 0.00124), confirming the increase in the stress-associated signature after chemotherapy.

### Stress-associated state is subclonally enriched during chemotherapy

**Fig. 4.**
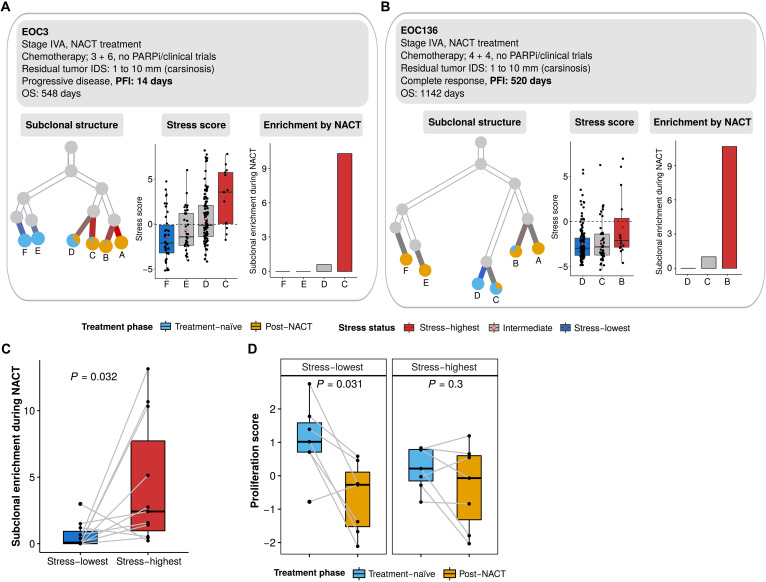
Inferred CNA and subclonal analysis reveals enrichment of the stress state during chemotherapy. (**A**) Inferred clonality tree (left), subclonal stress score (middle), and subclonal enrichment during NACT (right) of a representative patient (EOC3) with progressive disease and short PFI (PFI = 14 days). Only subclones that existed in the treatment-naïve samples are included in the subclonal stress score and subclonal enrichment analysis. The subclonal enrichment is measured by the ratio of the relative abundance of post-NACT cells against the relative abundance of treatment-naïve cells. PARPi, PARP inhibitor. (**B**) Inferred clonality tree (left), subclonal stress score (middle), and subclonal enrichment during NACT (right) of a representative patient (EOC136) with progressive disease and long PFI (PFI = 520 days). (**C**) Boxplot showing the enrichment of the stress-highest (red) and stress-lowest (blue) CNA subclones during NACT. Only subclones existing in treatment naïve samples (paired Wilcoxon rank-sum test, *P* = 0.032) were included. Each dot represents a CNA subclone. (**D**) Boxplots showing the proliferation score of the stress-highest (left; paired Wilcoxon rank-sum test, *P* = 0.031) and stress-lowest (right; paired Wilcoxon rank-sum test, *P* = 0.3) CNA subclones before and after chemotherapy. Each dot represents a CNA subclone.

To assess the effect of subclonal variation on the level of the stress-associated state, we used scRNA-seq data estimated copy number alteration (CNA) profiles to infer the subclonal structure of each patient (*30*). The subclonal CNA profiles inferred from scRNA-seq data had good concordance with subclonal CNA profiles obtained from the bulk whole-genome sequencing data from the same patients (Spearman’s correlation coefficient of 0.44 to 0.81; fig. S4A). Figure 4 (A and B) shows the inferred CNA subclonal structure of two representative patients: patient EOC3 with progressive disease and a PFI of only 14 days and patient EOC136 with complete response and a long PFI of 520 days. Both received standard NACT, had carcinosis after IDS, and neither participated in clinical trials nor received bevacizumab maintenance treatment. The subclones in EOC3 had generally higher stress scores than EOC136 in treatment-naïve samples, whereas the subclonal distances were longer for EOC3, indicating that, unexpectedly, the poor-response patient had lower level of genetic heterogeneity. In both patients, the subclones with higher stress scores in treatment-naïve samples were expanded more than low-stress subclones after chemotherapy (Fig. 4, A and B). The inferred CNA subclonality trees for all the 11 patients are shown in fig. S4B. Each patient had four to eight subclones, of which 12.5 to 100% were shared between each treatment-naïve and post-NACT sample pair. The four patients (EOC349, EOC540, EOC733, and EOC87) with all subclones shared between treatment-naïve and post-NACT samples had a median PFI of 1.99 months, indicating the limited efficacy of chemotherapy on these patients.

The inferred subclones showed significant differences in their stress scores in most patients (fig. S4B), which implies that the stress-associated state is at least partially driven by heritable differences across the subclones. Across the 11 patients studied, the subclones with the highest stress scores in treatment-naïve samples were significantly more expanded during chemotherapy when compared with the lowest-stress subclones (Fig. 4C). While the proliferation scores of the highest stress subclones remained similar, the proliferation scores of the lowest stress subclones dropped significantly after chemotherapy (Fig. 4D). This suggests that the lower ability to maintain or recover proliferation following chemotherapy contributes to the loss of stress-lowest subclones during chemotherapy. In summary, the preexisting stress-associated state offers a selective advantage to cancer cells during chemotherapy, explained by more inert proliferation when compared to stress-low subclones.

### Stress-associated transcriptional profile predicts poor prognosis in HGSOC

To investigate whether the stress-related transcriptional profile also promotes chemoresistance in treatment-naïve tumors on the patient level, we used TCGA deconvoluted bulk RNA-seq and clinical data from 271 patients (*5*, *31*). Of these, 86 patients were identified as stress-high and 144 as stress-low based on their stress scores (fig. S5A). We confirmed the high/low stress state using reverse-phase protein array data, which showed that the levels of phosphorylated c-Jun (CJUN_pS73, *P* = 0.0035) and its upstream kinase, phospho–c-Jun N-terminal kinase (JNK) (JNK_pT183Y185, *P* = 0.00077) and phosphorylated p38-α (P38_pT180Y182, *P* = 0.017), were significantly higher in the stress-high tumors compared to stress-low tumors (fig. S5B).

**Fig. 5.**
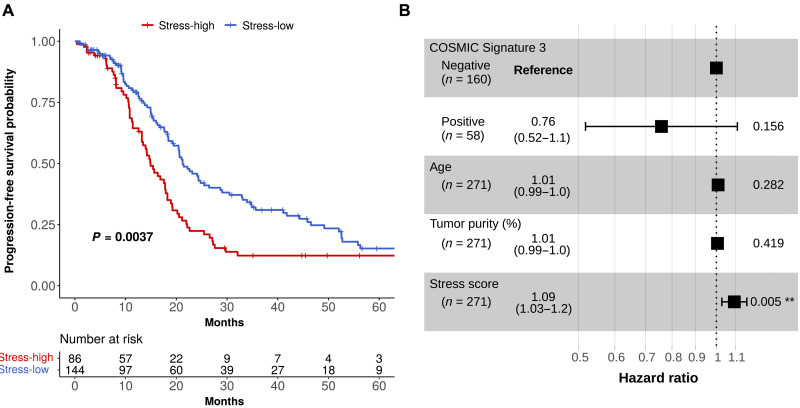
Stress-associated transcriptional profile predicts poor survival in HGSOC. (**A**) Stress-high and stress-low Kaplan-Meier curves on PFS for stress-high and stress-low patients (log-rank test, *P* = 0.0037) from the TCGA cohort. The number of patients at risk is listed below the survival curves for each time point. (**B**) Forest plot showing hazard ratios, their confidence intervals, and *P* values based on a multivariate Cox proportional hazards regression model testing whether PFS relates to COSMIC Signature 3 status, age at diagnosis, tumor purity, and stress score. **: 0.001-0.01.

Kaplan-Meier survival analysis indicated that patients with stress-high tumors at diagnosis have significantly shorter progression-free survival (PFS) time (log-rank test, *P* = 0.0037; Fig. 5A). The median PFSs in stress-high and stress-low groups were 14.9 and 21.2 months, respectively. HRD is a known prognostic factor for HGSOC (*32*). Thus, we tested whether the stress-associated state can be explained by COSMIC Signature 3 (COSMIC_Sig3), which is associated with HRD (*33*). As shown in fig. S5C, COSMIC_Sig3 was not found enriched in stress-high or stress-low patients (Fisher’s exact test, *P* = 0.31). Furthermore, multivariate Cox regression analysis showed that the stress score was significantly associated with short PFS (*P* = 0.005; Fig. 5B) independently of the effect of COSMIC_Sig3 status, age, or tumor purity. Thus, these results demonstrate that the stress-related transcriptional profile preexists in the treatment-naive tumors, and it is an independent predictor for poorly responding patients with HGSOC.

### Inflammatory stroma correlates with stress-associated cancer cells

Increased expression of proinflammatory cytokines, such as *IL6* and *TNF*, in the stress-associated cancer cell population suggests that these cells could have a substantial contribution to paracrine signaling. Therefore, we set out to analyze whether stress-associated state in cancer cells was reflected in differences of TME composition and potential interactions therein.

**Fig. 6.**
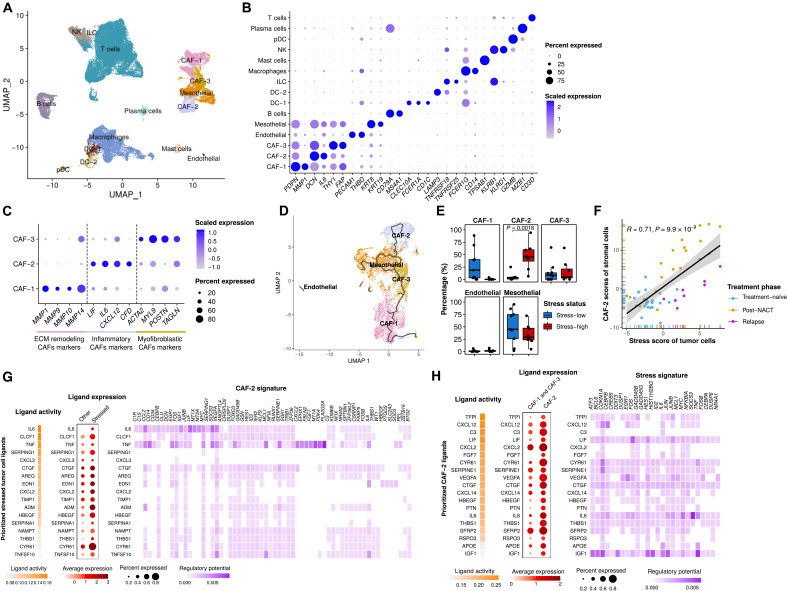
Interactions between inflammatory stroma and stress-associated cancer cells. (**A**) UMAP plot of stromal and immune cells, colored by cell type. (**B**) Dot plot showing the relative expression of acknowledged stromal and immune cell subtype markers. The color intensity scale reflects the average gene expression, and the size scale indicates the percentage of cells expressing the gene within that cell type. (**C**) Dot plot showing the expression of selected marker genes of CAF subtypes. ECM, extracellular matrix. (**D**) UMAP plot of stromal cells, colored by cell type. The trajectory learned by Monocle3 is displayed. (**E**) Boxplots showing the fractional differences (Wilcoxon rank-sum test) of identified stromal subtypes between stress-high (red) and stress-low (blue) tumors. Each dot represents a tumor sample. All differences with FDR-adjusted *P* < 0.05 are indicated. (**F**) Scatter plot showing the correlation between the tumor compartment stress score and the stromal compartment CAF-2 scores in HERCULES cohort. Each dot represents a sample, colored by treatment phase. (**G**) Heatmaps and dot plots showing the activity (left), expression (middle), and regulatory potential (right) of the prioritized ligands in stressed cancer cells that drive the phenotype of the inflammatory stroma (CAF-2). (**H**) Heatmaps and dot plots showing the activity (left), expression (middle), and regulatory potential (right) of the prioritized ligands in inflammatory stroma (CAF-2) that drive the stress signature in the stressed cancer cells.

We identified 10 immune and 5 stromal cell types based on the expression of canonical markers (Fig. 6, A and B): B cells, two types of dendritic cells (DCs), innate lymphoid cells (ILCs), macrophages, mast cells, natural killer (NK) cells, plasmacytoid DCs, plasma cells, T cells, endothelial cells, mesothelial cells, and three types of cancer-associated fibroblasts (CAFs; Fig. 6C). While none of the major immune cell types showed substantial proportional differences between stress-high and stress-low samples (fig. S6A), we set out to analyze cell state differences of the most prevalent immune cell types. Projection of T cells into a reference atlas (fig. S6, B and C) (*34*) suggested a decrease in CD8^+^ effector memory T cells and an increase of “precursor exhausted” T cells in stress-high samples (fig. S6D). In addition, macrophages in stress-high samples exhibited significantly higher expression of immunosuppressive features (*C1QA*, *C1QB*, *C1QC*, *APOE*, and *TREM2*) (fig. S6E) ((*14*, *35*), wherein *TREM2* is functionally associated with T cell exhaustion (*36*). Together, the analyses suggest that although the cell type prevalence in immune TME is not connected with stress-associated cancer cell state per se, the stress-high samples show a shift toward compromised tumor immunity.

In line with studies from other solid cancers, HGSOC tumors contain specialized CAF subpopulations with distinct functional markers: CAF-1–expressing matrix metalloproteinases (MMPs), CAF-2–expressing inflammatory CAF (iCAF) markers *IL6*, *CXCL12*, and *LIF* (*37*), and CAF-3–expressing markers of myofibroblast identity (Fig. 6C). Trajectory analysis to explore the relations between stromal cell types shows that iCAF and CAF-1 populations form separate branches that are joined via CAF-3 and mesothelial cells (Fig. 6D). Among the stromal cell populations, only iCAFs were significantly enriched in stress-high tumors (Fig. 6E), and their markers were also strongly associated with cancer stress scores in bulk RNA-seq data (Fig. 6F). Ligand-receptor analysis to probe for potential interactions revealed that, in particular, *TNF* and its downstream effector *IL6* from stress-high cancer cells have a strong regulatory potential to induce the inflammatory phenotype of CAFs (Fig. 6G and fig. S6F). This indicates that in NACT-treated ovarian cancer, *TNF/IL6* drives the iCAF phenotype rather than *IL1B*, which has a leading role in promoting the iCAF phenotype in pancreatic cancer (*38*). In response, iCAFs produce a wide array of ligands with rich regulatory potential to activate stress-associated signature within cancer cells, including both *IL6* and *TNF* to promote a paracrine feed-forward loop (Fig. 6H and fig. S6G). Our results suggest iCAFs as the main cell type expressing *IL6*, *CXCL12*, and *LIF* in the tumor milieu, wherein these ligands promote immunosuppressive changes, such as macrophage polarization, toward the M2 phenotype (*39*).

In summary, we found that stress-associated cancer cells strongly associate with presence of iCAFs within the TME and a shift toward immunocompromised states within macrophages and CD8^+^ T cells. The proinflammatory signaling molecules expressed by stress-associated cancer cells and iCAFs have the potential to promote paracrine feed-forward loops that can further induce these cell states. Targeting this signaling could be important, especially when chemotherapy is combined with immunotherapy, wherein ligands from iCAFs and stress-associated cancer cells may limit the chemotherapy-induced boost in the antitumor immune response. Our results of stress-associated cancer cells converge subclonal enrichment of cell state with feed-forward, immune suppressive paracrine signaling and offer both biomarkers and targets for novel combinatorial treatments.

## DISCUSSION

Approximately half of the patients with HGSOC do not have HRD tumors and lack durable responses to either chemotherapy or PARP inhibitors, leading to short survival. To address this unmet clinical need, we characterized nongenetic mechanisms of chemoresistance in a poorly responding patient cohort. Our novel single-cell transcriptomics analysis approach on 22 paired treatment-naïve and post-NACT HGSOC specimens from 11 patients revealed a consistent increase in a stress-associated state upon treatment. This finding is in line with a smaller study performed with NanoString (*40*).

We independently validated the expression of the core stress response genes by RNA-ISH of matched nondissociated tissue sections, hence confirming that the signal we detect is not a dissociation artifact as seen in previous scRNA-seq studies (*27*, *41*). The stress-associated state distills core acute stress response by IEGs with inflammatory prosurvival signaling by NF-κB targets, as well as key regulators of EMT and stemness to protect cancer cells from chemotherapy. These cells resist apoptosis (*BCL6*) and can boost DNA repair via increased *ATF3*, which stabilizes the major DNA damage kinase ataxia telangiectasia mutated (*42*).

Our results showed that the proportion of proliferative cell population in treatment-naïve samples decreased from an average of 14% to an average of 3% in the post-NACT samples. Thus, we demonstrate that even in our poorly responding patient cohort, where the median PFI was 4.2 months and only three patients achieved response evaluation criteria in solid tumors (RECIST) complete response, chemotherapy has a fundamental impact on the phenotype of cancer cells. This implies that the chemoresistance mechanisms driving poor clinical response are not related to platinum uptake or efflux but rather to preexisting and induced cellular states.

We showed that chemotherapy reduces the low-stress subclones efficiently, at least partially due to the significantly reduced proliferation levels of low-stress subclones, leaving behind a higher proportion of the cells in subclones with initially increased transcriptomic stress response. The subclonal differences between treatment-naïve and post-NACT samples are not deterministic but rather slightly bias the cells toward the stress-associated state, analogous to what was shown for the cellular states of untreated glioblastoma specimens on the subclonal level (*16*). A previous analysis of paired pre- and post-NACT samples of triple-negative breast cancer found subclonal evolution to shape the genetic composition of tumors but failed to detect any shared definitive expression patterns to be subclonally enriched during chemotherapy (*13*). Thus, our results provide the first evidence of parallel subclonal selection of a defined transcriptional phenotype during chemotherapy in human tumors. Both the subclonal and patient level analyses strongly suggest that the preexisting stress-associated state primes the cancer cells to endure chemoresistance.

We did not detect recurrent genomic changes that would explain the subclonal differences in the stress-associated state, suggesting that they are either highly multigenic or based on epigenetic features or genomic aberrations other than CNAs. It remains to be assessed whether subclonal differences directly affect the level of intrinsic stress based on, for instance, metabolic features or rather modify the level of response to potential environmental stressors, such as hypoxia, lack of nutrition, or excess inflammatory signaling from their microenvironment.

Tumor stroma has been suggested to play a key role in chemoresistance of many cancers, including HGSOC, and increased tumor-stroma proportion at initial diagnosis of HGSOC associates with chemoresistance (*43*, *44*). Here, we found that, specifically, the *IL6* high iCAFs co-occur with the stress-associated cancer cells, complementing a recent spatial transcriptomics study of pancreatic ductal adenocarcinoma (*45*). A chemoresistant role for TME-derived interleukin-6 (IL6) is further supported by findings where increased IL6 in peritoneal fluid (*46*), ascites (*40*), or blood plasma (*47*) associate with worse prognosis of patients with HGSOC. Ligand-receptor analysis suggests that paracrine signaling is amplifying the stress response by a feed-forward loop in both cancer cells and iCAFs. This paracrine signaling is highly boosted by systemic platinum-taxane chemotherapy that not only causes extreme genotoxic and mitotic stress in cancer cells but also induces stress response in the nonproliferating stroma (*43*).

The stress-induced adaptation pattern we observed may offer avenues for therapeutic intervention. As direct targeting of the core immediate-early genes by mitogen-activated protein kinase/extracellular signal–regulated kinase pathway inhibitors is unlikely to work (*48*), targeting the inflammatory paracrine signaling may provide the most promising approach for combinatorial therapies. The nonproliferating TME is not under selective evolutionary pressure, reducing the risk of treatment resistance. Among the current treatment regimens, the antiangiogenic bevacizumab may hold promise for the *IL6*-expressing stress-high tumors, as increased plasma levels of IL6 are indicative of bevacizumab sensitivity in HGSOC (*47*). Furthermore, antibodies against IL6, TNF, LIF, CXCL12, or their receptors, some of which are already in clinical use to treat inflammatory diseases, have shown initially promising results in preclinical models when combined with platinum chemotherapy (*49*–*52*). In addition, the regulators up- and downstream of *IL6*, namely, *STAT3* and Toll-like receptors, respectively, have been successfully targeted in resistant cancer models (*53*, *54*). Targeting these inflammatory cytokines has also shown promising results in combination with immunotherapies [e.g., in ovarian cancer models (*55*, *56*)] This implies that the stress response may provide cancer cells with resistance against a wide array of treatments, from chemotherapy to targeted therapies and immunotherapies, and thus provide targets for a generalized strategy to oppose resistance in cancer.

We have identified a stress-associated state that distills acute stress response with paracrine inflammatory signaling to provide cancer cells with adaptation, promoting chemoresistance on both subclone and patient level. Overall, our results support a combination of induced and selective processes to explain chemotherapy-induced transcriptomic changes as suggested in (*13*), modified by both subtle genetic differences and changes in the TME signals. Furthermore, the identification of stress signature opens avenues for combinatorial drug testing in preclinical models that maintain both subclonal heterogeneity and paracrine tumor-stromal signaling. As many drugs targeting inflammatory effectors are already in clinical use for other indications, they may offer a realistic option for safe combinatorial therapies with a wide array of currently used oncological drugs to restrain the broadly adaptive stress response of tumors.

## MATERIALS AND METHODS

### Human participants

All patients participating in the study provided written informed consent. The study and the use of all clinical materials have been approved by the Ethics Committee of the Hospital District of Southwest Finland (ETMK) under decision number EMTK: 145/1801/2015.

The clinical specimens used in the study represent several understudied aspects of HGSOC that are poorly represented in existing cohorts of clinical specimens, such as TCGA (*5*). Contrary to TCGA data, all our paired samples were collected from intra-abdominal, peritoneal, and omental metastases, thus representing cancer cell populations with proven metastatic potential. The material was from solid tumors, containing potentially chemoprotective stromal TME, which is missing from the more broadly available ascites samples. Our cohort also included low purity tumors that may represent a distinct, poor prognosis phenotype of HGSOC, which are missing from most genomic analyses.

### scRNA-seq sample preparation

Prospective HGSOC tumor specimens were collected from 11 patients at the time of laparoscopy and IDS. Detailed clinical information is shown in Table 1. Immediately after surgery, the specimens were incubated overnight in a mixture of collagenase and hyaluronidase (Department of Pathology, University of Turku) to obtain single-cell suspensions. For samples specified in Table 1, single-cell suspensions were frozen in STEM-CELLBANKER DMSO-FREE solution (#11897F, AMSBIO) and thawed in culture medium immediately before processing for scRNA-seq. The viability of the frozen single-cell suspensions ranged from 65 to 94% after thawing, with a median of 80%. scRNA-seq libraries were prepared with the Chromium Single-Cell 3′ Reagent Kit v. 2.0 (10x Genomics) and sequenced on Illumina HiSeq 4000 (Jussi Taipale Lab, Karolinska Institute, Sweden), HiSeq 2500, and NovaSeq 6000 instruments (Sequencing Unit of the Institute for Molecular Medicine Finland, Finland).

### Preprocessing scRNA-seq data

The Cell Ranger software suite (version 3.1.0) was used to perform sample demultiplexing, alignment, barcode processing, and UMI quantification. The reference index was built upon the GRCh38.d1.vd1 reference genome with GENCODE v25 annotation. We applied a three-step filtering approach to filter out low-quality cells. In the first steps, we excluded cells expressing any combinations of *PAX8*, *DCN*, and *PTPRC* to remove potential doublets and removed cells with above 15% UMI counts originating from mitochondrial genes. Then, we used the shared nearest neighbor (SNN) modularity optimization–based clustering from Seurat v3 (*15*) for initial clustering. Three major cell types were revealed on the basis of acknowledged markers: epithelial cancer cells (*WFDC2*, *PAX8*, and *EPCAM*), stromal cells (*COL1A2*, *FGFR1*, and *DCN*), and immune cells (*CD79A*, *FCER1G*, and *PTPRC*).

In the second filtering step, we quantified the quality measures of each cell using Seurat v3 (*15*). We estimated the cutoffs for each quality measure in each cell type based on its bimodal distribution (fig. S1B) and then used four criteria for quality control: (i) the number of reads above 8192 for cancer cell, 4096 for stromal cells, and 2896 for immune cells; (ii) the number of UMI counts above 4075 for cancer cells, 2048 for stromal cells, and 1024 for immune cells; (iii) the number of detected genes above 1552 for cancer cells, 1024 for stromal cells, and 512 for immune cells; and (iv) the percentage of UMI counts originating from mitochondrial genes below 12 for cancer cells and 7.5 for stromal and immune cells. Third, we filtered out epithelial cells with inferred CNA profiles that clustered together with stromal cells.

### Modeling and clustering scRNA-seq data of cancer cells using PRIMUS

PRIMUS models the observed single-cell expression profiles as a mixture of latent phenotypic transcriptional profiles and nuisance expression profiles following a Poisson distribution

$$Y_{j,i}\sim \text{Poisson}\;((\sum_{l=1}^{r}(X_{j,l}D_{l,i})+\sum_{c=1}^{k}(Z_{j,c}C_{c,i}))G_{i})$$ (1)

where *l* = 1,2, …, *r* runs over the *r* nuisance factors and *c* = 1,2, …, *k* runs over *k* latent phenotypic clusters. *Y*_*j*,*i*_ denotes the observed UMI counts of gene *j* in the *i*th cell, *X*_*j*,*l*_ denotes the expression profile centroid of gene *j* specific to nuisance factor *l*, *D*_*l*,*i*_ denotes the design coefficient of the *l*th nuisance factor in the *i*th cell, *Z*_*j*,*c*_ denotes the cluster *c* expression profile centroid at gene *j*, *C*_*c*,*i*_ ∈ {0,1} is an indicator of whether the *i*th cell belongs to the cluster *c*, and *G_i_* is a cell-specific scaling factor.

A linear model, such as in Eq. 1, is appropriate when the action of nuisance signals and the biological phenotypic signals can be considered additive. This occurs when the processes are parallel or their action is nonoverlapping, e.g., when specific pathways (or the genes within) are controlled by the patient-specific component and others are controlled by the cell state. The use of a stochastic model permits natural variation between cells.

We highlight that while the underlying components are Poissonian, the observed counts *Y*_*j*,*i*_ is a mixture of Poisson-distributed factors with unequal rates, as specific in Eq. 1, which results in an overdispersed data distribution. The Poisson model is also well suited for capturing random RNA dropout (*57*, *58*), which is commonly observed in scRNA-seq data (*59*).

Given the observations *Y*_*j*,*i*_, known nuisances *D*_*l*, *i*_, known scaling factors *G_i_*, and the number of latent clusters *k*, we can estimate the latent nuisance expression centroids *X*_*j*,*l*_, latent expression centroids *Z*_*j*,*c*_, and the latent cluster memberships *C*_*c*,*i*_ using an expectation-maximization (EM) algorithm (*60*). The EM algorithm is constructed on the latent variables *Z*_*X*_*j*,*l*,*i*__ ∼ Poisson(*X*_*j*,*l*_*D*_*l*,*i*_*G_i_*) and *Z*_*Z*_*j*,*c*,*i*__ ∼ Poisson(*Z*_*j*,*c*_*C*_*c*,*i*_*G_i_*), which are the nuisance and cleaned contributions to the expression, respectively. The parameter set θ = (*X*_*j*,*l*_, *Z*_*j*,*c*_, *C*_*c*,*i*_) was estimated in two stages: First, the expression centroids *X*_*j*,*l*_ and *Z*_*j*,*c*_ can be estimated given *Y*_*j*,*i*_, *D*_*l*,*i*_, *C*_*c*,*i*_, and *G_i_*; second, the cluster membership *C*_*c*,*i*_ can be updated given *Y*_*j*,*i*_, *X*_*j*,*l*_, *D*_*l*,*i*_, *Z*_*j*,*c*_, and *G_i_*. Given *Y*_*j*,*i*_, *D*_*l*,*i*_, *G_i_*, and the estimated *X*_*j*,*l*_, we further computed ${\tilde{Z}}_{j,i}$, the denoised expression of gene *j* in the *i*th cell by solving $Y_{j,i}\sim \text{Poisson}\;((\sum_{l=1}^{r}(X_{j,l}D_{l,i})+{\tilde{Z}}_{j,i})G_{i})$ for ${\tilde{Z}}_{j,i}$. See the Supplementary Materials for details.

To select the optimal *k*, we fitted PRIMUS for *k* = 1,2, …,25 with 10 different random initial parameter sets for each *k*, and *k* = 12 was selected on the basis of BIC (fig. S2A). We then ran the EM procedure with 200 random initializations for *k* = 12, the maximum likelihood estimates of *X*_*j*,*l*_ and *Z*_*j*,*c*_, and *C*_*c*,*i*_ and ${\tilde{Z}}_{j,i}$ were used for downstream analysis. The model selection process also acts as a regularizer for penalizing clusters that are solely correlating with the modeled nuisance factors. This tends to make the method to favor solutions where the effect of the confounding factors is completely eliminated in case of overlap.

### Simulation of scRNA-seq datasets

We simulated scRNA-seq datasets using the splatPop model from the R package splatter (*24*, *25*). Provided with genotype information for a population, splatPop models expression quantitative trait loci (eQTL) effects and simulates gene counts for single cells for individuals in the population. Following the suggested pipeline (https://bioconductor.org/packages/release/bioc/vignettes/splatter/inst/doc/splatPop.html), we used the mockVCF function to generate mock variant call format (vcf) files for 20,000 single-nucleotide polymorphisms in six samples, the mockBulkeQTL function to generated mock eQTL mapping results for 5000 genes, and the mockBulkMatrix function to generate mock bulk expression data of 5000 genes for a population with 100 samples, with the default parameters. We next estimated the simulation parameters for the eQTL population simulation from the generated mock eQTL mapping results and bulk expression data using the splatPopEstimate function. Last, we used the splatPopSimulate function to simulate scRNA-seq count data using the mock vcf files and the estimated parameters for six samples and five cell groups under three scenarios (table S1): (i) All six samples contain the five cell groups (3000 cells and 5000 genes); (ii) each sample only contains a subset of cell groups, three pairs of samples had no cell types in common, and there was one sample-specific cell group (1400 cells and 5000 genes); (iii) the same setting with scenario ii but with unbalanced cell numbers in each sample (from 20 to 2000). We simulated 20 random datasets from each scenario for benchmarking.

### Human pancreatic datasets

We obtained five human pancreatic datasets and the corresponding cell type annotations from https://github.com/JinmiaoChenLab/Batch-effect-removal-benchmarking/tree/master/Data/dataset4 (*61*). This dataset contains 14,767 cells in total with 15,558 genes for 15 different cell types and 45 samples from five studies (*62*–*66*). The sample labels were collected from GSE84133 (*62*), GSE85241 (*63*), E-MTAB-5061 (*64*), GSE83139 (*65*), and GSE81608 (*66*), respectively. We randomly sampled 80% of the cells 20 times and assessed the cell type identification performance of PRIMUS and other methods on the subsampled datasets.

### Comparison of PRIMUS to other methods

We compared PRIMUS to five commonly used single-cell data integration methods [Seurat v3 (*15*), Harmony (*19*), LIGER (*18*), mnnCorrect (*23*), and fastMNN (*23*)] and three bulk data integration methods [ComBat (*21*), ComBat-seq (*22*), and limma (*20*)].

#### PRIMUS

PRIMUS takes the raw count matrix, design of nuisance factors, and scaling factors as inputs. For the simulated datasets, the nuisance factors were the sample labels, and the scaling factors were estimated using the logNormCounts function from scater R package (version 1.20.0) (*67*) following the splatPop (*24*, *25*) simulation tutorial. For the pancreatic datasets, the nuisance factors were the sample labels, and the scaling factors were estimated using the prism-gain function from the PRISM package (*31*). The number of clusters *k* was set to the same as the number of cell groups/types, and the maximum number of iterations for EM procedure was set to 200.

#### Seurat v3

We ran Seurat v3.2.3 (*15*) as described in Seurat’s integration tutorial (https://satijalab.org/seurat/articles/integration_introduction.html) for the pancreatic datasets and simulation scenarios i and ii datasets. Sample_6 in scenario iii contained only 20 cells, and sample ICRH76 from the pancreatic datasets contains only 19 cells, which were too few for Seurat v3 to perform integration, so Seurat v3 was not run on datasets from scenario iii and the pancreatic datasets. We performed clustering on the first 30 principal components (PCs) for the integrated pancreatic datasets and on the first 20 PCs for the integrated simulated datasets, using the FindNeighbors and FindClusters functions.

#### Harmony

We ran Harmony (*19*) according to its online tutorial (https://github.com/immunogenomics/harmony). We ran Harmony with default parameters on the first 30 PCs for the pancreatic datasets and the first 20 PCs for the simulated datasets and obtained the corrected PC embeddings. We used FindNeighbors and FindClusters functions from Seurat v3 (*15*) to run clustering on Harmony-corrected PC embeddings.

#### LIGER

We ran LIGER (rliger package, version 1.0.0) (*18*) with the default parameters (*k* = 20, λ = 5) as suggested in the integration tutorial (http://htmlpreview.github.io/?https://github.com/welch-lab/liger/blob/master/vignettes/Integrating_multi_scRNA_data.html). We set *k* = 10 for the pancreatic datasets as the smallest sample contains only 19 cells.

#### mnnCorrect and fastMNN

We followed the tutorial (http://bioconductor.org/packages/devel/bioc/vignettes/batchelor/inst/doc/correction.html) to run the mnnCorrect and the fastMNN functions from the batchelor package (version 1.8.0) (*23*). We used the top 5000 and top 1000 highly variable genes (HVGs) for correction for the pancreatic datasets and the simulated datasets, respectively. All other parameters were kept as default values.

#### ComBat and ComBat-seq

ComBat (*21*) was initially designed to remove batch effects in microarray data, and ComBat-seq (*22*) is an extension of ComBat to address batch effects in bulk RNA-seq data. We ran ComBat and ComBat-seq using the implementation in the R package sva (version 3.40.0) (*68*) with default parameters.

#### limma

We followed the user guide https://www.bioconductor.org/packages/devel/bioc/vignettes/limma/inst/doc/usersguide.pdf to run limma (version 3.50.0) (20). As limma expects normalized and log-transformed data as input, we first normalized the raw counts using the “LogNormalize” method from the NormalizeData function in Seurat v3 (*15*) and ran limma with the normalized data using the removeBatchEffect with default parameters. 

For mnnCorrect, fastMNN, ComBat, ComBat-seq, and limma, which do not have recommended clustering approaches in their online tutorials, we applied the Louvain clustering (*69*) implemented in LIGER (*18*) on their integration outputs. For all methods except for PRIMUS, the clustering was run with the resolution parameter ranging from 0.01 to 5, and the outputs with the number of clusters the same as the number of cell groups/types were used.

We computed the adjusted rand index (ARI) (*70*) to compare the cell group/type labels with the computed cluster labels for the simulated and pancreatic datasets. We used the adjustedRandIndex from the mclust R package (version 5.4.7) (*71*) to compute ARI.

### Differential expression analysis for cancer cells

We used an LRT to perform the differential expression (DE) analysis controlling for the nuisance factors. Let *Y*_*j*, *i*_ denote the observed UMI count of gene *j* in the *i*th cell, $\mu_{j,i}=(\sum_{l=1}^{r}(X_{j,l}D_{l,i})+\sum_{c=1}^{k}(Z_{j,c}C_{c,i}))G_{i}$ denotes the predicted mean expression rate of gene *j* for the *i*th cell based on the estimated model parameters *X*_*j*,*l*_, *Z*_*j*,*c*_, and *C*_*c*,*i*_. The DE between group *g*_1_ and group *g*_2_ for the gene *j* can be assessed by testing the alternative hypothesis *H*_A_ : *Z*_*j*,*g*_1__ ≠ *Z*_*j*,*g*_2__ against the null hypothesis *H*_0_ : *Z*_*j*,*g*_1__ = *Z*_*j*,*g*_2__. For the former, the likelihood is that attained at the maximum-likelihood estimate (MLE) ${\hat{\mu }}_{j,i}$, while for the latter, the model is refitted giving ${\bar{\mu }}_{j,i}$, the MLE under *H*_0_. The logarithmic LRT statistic for gene *j* is

$${\text{LRT}}_{j}=\sum_{i\in I_{1}\cup I_{2}}(Y_{j,i}\text{log}{\hat{\mu }}_{j,i}-{\hat{\mu }}_{j,i})-\sum_{i\in I_{1}\cup I_{2}}(Y_{j,i}\text{log}{\bar{\mu }}_{j,i}-{\bar{\mu }}_{j,i})$$

where *I*_1_and *I*_2_ denote the indices for the samples in *g*_1_and *g*_2_, respectively. A *P* value for the gene *j* to be differentially expressed between group *g*_1_ and group *g*_2_ can be computed as the probability to the right of the −2LRT*_j_* for the chi-squared distribution with degrees of freedom is equal to the difference in number of parameters, i.e., 1.

### Identification of coexpressed gene communities

Coexpressed gene communities were identified as follows: (i) We conducted DE analysis between each pair of cell clusters, resulting in 66 comparisons. The top 1000 most significant LRT genes with an FDR of <0.01 were selected in each comparison, and this resulted in a total of 4742 genes; (ii) the Pearson correlations between these 4742 genes were computed using the LRTs from all 66 comparisons. Correlations with ρ > 0.8 and *P* < 0.01 were used to build a gene network; (iii) we detected 916 communities in the network using the Walktrap community finding algorithm with step equals to 3 (*72*), and the 10 communities consisting of more than 30 genes were retained for further analysis; (iv) let *V* be the genes in a community, *c_j_* be the coreness of gene *j*, and *n*_max_ be the number of genes with the maximum coreness (max_*j* ∈ *V*_*c_j_*, degeneracy) in that community. If *n*_max_ > 30, then the genes with *c_j_* = max_*j* ∈ *V*_*c_j_* were retained; otherwise, we retained the top 30 genes ranked by coreness; (v) gene set overrepresentation analysis was performed for the remaining genes in each community using the ConsensusPathDB (*26*). We further reduced the redundancy of each gene community with number of genes above 20 by applying the following filters: (i) Only genes overlapped with significantly overrepresented gene sets (FDR < 0.05, size <500) were kept; and (ii) biclustering was applied on the binary matrix of the presence/absence of each gene in each significantly overrepresented gene set using the R package blockcluster (version 4.4.3) (*73*), and the gene clusters that have less than 3% presence in any of the gene set clusters were excluded. After filtering, the numbers of genes per community were between 11 and 106. The genes in each community are listed in table S2.

### Quantification of stress scores and proliferation scores from RNA-seq data

We defined the stress score as the gene set enrichment score of our identified stress-associated gene signature in individual cells and samples, which was computed using Single sample Gene Set Enrichment analysis (ssGSEA) (*74*). Samples with permutation test *P* value below 0.05 by permutation test were considered stress-high, while samples with *P* value above 0.5 were considered stress-low.

Similarly, we quantified the proliferation score as the gene set enrichment score of the proliferative DNA repair gene signature in individual cells and samples using ssGSEA (*74*).

### RNA-ISH and imaging

RNA-ISH was performed on fresh 3-μm formalin-fixed paraffin-embedded tissue sections using the RNAscope Multiplex Fluorescent Reagent Kit version 2 for target detection (#323100, Advanced Cell Diagnostics) according to the manual. Briefly, tissue sections were baked for 1 hour at 60°C, then deparaffinized, and treated with hydrogen peroxide for 10 min at room temperature. Target retrieval was performed for 15 min at 98°C, followed by protease plus treatment for 15 min at 40°C. All RNAscope probes (tables S3 and S4) were hybridized for 2 hours at 40°C, followed by signal amplification, and development of horseradish peroxidase channels was performed according to the manual. TSA Plus fluorophores fluorescein (1:750 dilution), Cyanine 3 (1:1500 dilution), and Cyanine 5 (1:3000 dilution) (NEL744001KT, PerkinElmer) were used for signal detection. The sections were counterstained with 4′,6-diamidino-2-phenylindole (DAPI) and mounted with the ProLong Gold Antifade Mountant (P36930, Invitrogen). Images were generated using 3DHISTECH Pannoramic 250 Flash II digital slide scanner at the Genome Biology Unit supported by HiLIFE and the Faculty of Medicine, University of Helsinki, and Biocenter Finland. All samples were scanned using ×40 magnification with extended focus and seven focus levels.

### Quantitative analysis of whole-slide RNA-ISH images

We used CaseViewer (version 2.3.0, 3DHISTECH Ltd.) to read the MRXS immunofluorescence image and to separate its different channels into the DAPI staining, and fluorescein (FITC 38 HE), Cyanine 3 (TRITC 48 HE) and Cyanine 5 (Cy5) channels for gene expression quantification. CellProfiler (version 3.1.8) (*75*) was used for segmentation in the DAPI staining. The nondefault parameters that were determined experimentally were as follows: A typical diameter of 18 to 56 pixels, thresholding using adaptive Otsu’s method, clumped object detection and splitting using shape, and low-resolution speedups were disabled. The segmented objects were classified into cancer, immune, and stromal cells using the DAPI staining and its segmentation. For this, we extracted the area, the mean nucleus stain intensity, and the eccentricity of each segmented object. Subsequently, we trained a supervised quadratic classifier using different training sets of cells with the properties mentioned above and desired cell types. Since the cancer and immune cell morphology and intensity change from primary to interval samples and there are also some stromal cells hard to distinguish from small cancer cells, we trained multiple classifiers to obtain the highest classification accuracy per image. The classification results were visually assessed by a pathologist. Afterward, the classifier was used to predict the cell types using the computed features in untrained images. The quadratic classifier was implemented in MATLAB (version R2019b) and was trained with uniform class priors. We extracted spatial probability maps for each cell type from the quadratic classifier, which were then low pass–filtered in logarithmic space (probability product space) using a disk kernel of 100-pixel radius (cf. cell radius of ~20). This propagates the probability of classification to neighboring cells in the regions with large classification uncertainty, but for a cell exhibiting strong features of a particular type, its class will be unaffected.

Since some RNA signals are localized in the cytoplasm of the cells, we have expanded the segments of corresponding tumor, immune, and stromal nuclei to include the cellular cytoplasm. This expansion was performed by dilating the segments in the unlabeled space with a disk kernel with a radius size of 20, 5, and 5 pixels for the tumor, immune, and stromal classes, respectively. Ties were broken to the nearest segment. The parameters were tuned experimentally to account for the different sizes between the different cell types.

We reduced the cross-channel fluorescence bleed of Cy5, FITC, and TRITC staining by finding a suitable basis for the intensity data near the principal axes using power iteration. The fluorescence intensity signal was quantified using the negative response of a Laplacian of Gaussian filter with standard deviation of unity. The value was tuned manually, and the kernel width roughly corresponds to the diameter of an observed RNA spot in our images. This procedure filters out background variations and cellular autofluorescence, leaving intensity blobs of the specified size.

### Quantification of stress score from RNA-ISH data

To quantify the stress score using expression levels of the 10 stress-associated genes measured with RNA-ISH experiment, we performed the CCA between the RNA-ISH expression levels and the combination of treatment phase information and the scRNA-seq–derived stress score. The resulting first canonical component of the RNA-ISH quantifications, which is a linear combination of the expression levels of the 10 genes, was defined as the “RNA-ISH stress score.” The coefficients for each gene in the first canonical component of the RNA-ISH data are given in table S5.

To assess the significance of the correlation between the RNA-ISH and scRNA-seq stress scores and the difference between the treatment-naïve/post-NACT pairs in RNA-ISH stress scores, each of which is expected to have nonzero correlation by construction, the data were permuted 10^5^ times, and the analysis was applied on the permuted datasets to obtain empirical *P* values.

### Inference of CNA and clonal structure

The CNAs and subclones were inferred using inferCNV (version 1.4.0) (*30*) with the following parameters: “cutoff=0.1, denoise=TRUE, HMM=TRUE, hclust_method=‘ward.D2’, tumor_subcluster_partition_method=‘random_trees’, tumor_subcluster_pval=0.05, num_threads = 10.” We randomly sampled up to 150 stromal cells from each patient to serve as reference. We filtered out the subclones with less than five cells. The phylogenetic trees were generated using UPhyloplot2 (*76*).

### TME cell type annotation

Clustering of stromal and immune cells was performed using Seurat v3 (*15*). We selected the top 3000 HVGs using the FindVariableFeatures function with the method “vst.” The expression of those HVGs was centered and scaled using the ScaleData function with default parameters. We performed PC analysis on the scaled data, and the SNN modularity optimization–based clustering was conducted using the first 50 PCs with a resolution parameter of 3. Next, we performed cell type annotation using Scibet (*77*), a supervised cell type annotation tool, which can accurately predict cell identities regardless of technical factors or batch effect (*77*), as follows: (i) First, we predicted the cell type for each stromal and immune cell with a trained model provided by Scibet, which includes 30 major human cell types from 42 scRNA-seq datasets as the reference. (ii) Second, for each cell type identified in step i, we used the cells from the clusters, of which more than 75% cells belong to that cell type, to build a new reference set. (iii) Third, we annotated the remaining cells using SciBet with the reference set built in step ii. The cell type name was corrected manually in accordance with known gene markers: B cells (*CD79A*^+^ and *MS4A1*^+^), DC-1 (*CLEC10A*^+^, *FCER1A*^+^, and *CD1C*^+^), DC-2 (*LAMP*^+^), ILCs (*TNFRSF18*^+^ and *TNFRSF25*^+^), macrophages (*FCER1G*^+^), mast cells (*TPSAB1*^+^), NK cells (*KLRB1*^+^ and *KLRD1*^+^), plasmacytoid DCs (*GZMB*^+^), plasma cells (*MZB1*^+^), T cells (*CD3D*^+^), endothelial cells (*PECAM1*^+^ and *THBD*^+^), and mesothelial cells (*KRT8*^+^ and *KRT19*^+^). We identified CAFs as cell clusters that are positive for *FAP* and negative for cytokeratins (*KRT8*, *KRT18*, and *KRT19*). CAF subtypes were annotated on the basis of the markers: CAF-1 (*MMP1*^+^ and *MMP9*^+^), CAF-2 (*LIF*^+^, *IL6*^+^, *CXCL12*^+^, and *CFD*^+^), and CAF-3 (*ACTA2*^+^ and *MYL9*^+^).

### Trajectory analysis of stromal cells

To explore the relations of the identified stromal cell types, we constructed the cell trajectories using Monocle3 (version 1.0.0) (*78*). We removed the sample-specific variations and the effect of the percentage of the UMI counts originating from mitochondrial genes using PRIMUS before applying Monocle3. The denoised counts were log-transformed and projected into the first 30 PCs. We then computed the uniform manifold approximation and projection (UMAP) using the reduce_dimension function from Monocle3 with cosine distance, and the minimum distance was set to 0.3. We clustered the cells using the cluster_cells function with default parameters. Last, Monocle3 learned the trajectory graph using the learn_graph function with default parameters.

### DE analysis for TME cells

The identification of CAF subtype marker genes and the DE analysis between stress-high and stress-low samples for TME cells were conducted using the Seurat v3 (*15*) function FindMarkers using the negbinom test with the UMI counts, patient labels, library preparation method, and sequencing instruments as the latent variables.

### NicheNet analysis

NicheNet (*79*) was used to explore the cell to cell interactions between stressed cancer cells and iCAFs. We first calculated two sets of DEGs: CAF-2 versus CAF-1 and CAF-2 [DEG set1; log_2_ fold change (log_2_FC) > 0.25, adjusted *P* < 0.01, expressed in at least 25% of iCAFs] and stressed cancer cells versus other cancer cells (DEG set2; log_2_FC > 1, adjusted *P* < 0.01, expressed in at least 25% of stressed cancer cells). To identify which ligands produced by stressed cancer cells are driving the phenotype of CAF-2, we used the top 200 up-regulated genes in DEG set1 based on adjusted *P* value as gene set of interest. All genes expressed in at least 25% of CAF-2 were used as a background gene set. We required the potential ligands to be higher expressed in stressed cancer cells compared to other cancer cells (DEG set2) to narrow down the number of ligands to be evaluated. Similarly, we also identified the potential ligands produced by CAF-2 that are active in driving the stress signature in stressed cancer cells. The 35 genes in the stress signature were defined as the gene set of interest, and the background gene set included all genes expressed in at least 25% of stressed cancer cells. The potential ligands were higher expressed in CAF-2 compared to other CAFs (DEG set1). The lists of the DEG sets used in this analysis are provided in data S1 and S2.

### Bulk tumor expression data

We acquired 18 treatment-naïve versus post-NACT sample pairs and 8 primary-relapse sample pairs from 23 patients in the HERCULES cohort (http://project-hercules.eu/). The sample collection, data quality control, alignment, and quantification were performed as we have previously described (*31*).

TCGA RNA-seq data of ovarian serous cystadenocarcinoma (OV, illuminahiseq_rnaseqv2-RSEM_genes_normalized) was downloaded from the Broad Firehose (https://gdac.broadinstitute.org/), along with the clinical annotations. The primary tumors from 271 patients with advanced HGSOC (grade: G2 to G4, stage: IIIA to IV) and with PFS data available were included in our analysis. The proportions of tumor, stromal, and immune components and the cell type–specific expression profiles for HERCULES and TCGA samples were estimated using PRISM (*31*).

### TCGA reverse phase protein array data

The reverse phase protein array data (replicates-based normalization) for TCGA ovarian serous cystadenocarcinoma samples (TCGA-OV-L4) was downloaded from the Cancer Proteomics Atlas (https://tcpaportal.org/tcpa/download.html).

## Notes

View/request a protocol for this paper from *Bio-protocol*.
