# Supplementary material for: Longitudinal single-cell RNA-seq analysis reveals stress-promoted chemoresistance in metastatic ovarian cancer
Source: Sci Adv. 2022 Feb 23;8(8):eabm1831. doi: 10.1126/sciadv.abm1831 (PMC8865800; doi:10.1126/sciadv.abm1831)
Supplement: Supplementary file 1 — Supplementary Text Figs. S1 to S6 Tables S1 to S5 [file sciadv.abm1831_sm.pdf]

Science Advances
AAAS

Supplementary Materials for

Longitudinal single-cell RNA-seq analysis reveals stress-promoted chemoresistance in metastatic ovarian cancer

Kaiyang Zhang, Erdogan Pekcan Erkan, Sanaz Jamalzadeh, Jun Dai, Noora Andersson, Katja Kaipio, Tarja Lamminen, Naziha Mansuri, Kaisa Huhtinen, Olli Carpén, Sakari Hietanen, Jaana Oikkonen, Johanna Hynninen, Anni Virtanen, Antti Häkkinen, Sampsa Hautaniemi*, Anna Vähärautio*

*Corresponding author. Email: sampsa.hautaniemi@helsinki.fi (S.H.); anna.vaharautio@helsinki.fi (A.Vä.)

Published 23 February 2022, *Sci. Adv.* **8**, eabm1831 (2022)
DOI: 10.1126/sciadv.abm1831

The PDF file includes:

Supplementary Text
Figs. S1 to S6
Tables S1 to S5
Legends for data S1 and S2

Other Supplementary Material for this manuscript includes the following:

Data S1 and S2

Supplementary Text

PRIMUS model

Model structure. We assume each observed single cell expression profile is a mixture of a latent cellular states and nuisance expression profiles, and can be approximated by a Poisson distribution:

$$Y_{j,i} \sim Poisson\left(\left(\sum_{l=1}^{r}(X_{j,l}D_{l,i}) + \sum_{c=1}^{k}(Z_{j,c}C_{c,i})\right)G_i\right) \quad (1)$$

where $Y_{j,i}$ denotes the observed UMI counts of gene j in the i^{th}cell, $X_{j,l}$ denotes the expression profile centroid of gene j specific to nuisance factor l, $D_{l,i}$ denotes the design coefficient of the lth nuisance factor in the i^{th}cell, $Z_{j,c}$ denotes the cluster c expression profile centroid at gene j, $C_{c,i} \in \{0,1\}$ is an indicator of whether the i^{th}cell belongs to the cluster c, and G_i is a cell-specific scaling factor.

Parameter estimation. Given $Y_{j,i}$, $D_{l,i}$, G_i and the number of latent clusters k, we can estimate $X_{j,l}$, $Z_{j,c}$ and $C_{c,i}$ using an Expectation-Maximization (EM) algorithm *(60)*, which is constructed on the latent variables $Z_{X_{j,l,i}}$, $Z_{Z_{j,c,i}}$and the observations $Y_{j,i}$:

$$\begin{aligned} Z_{X_{j,l,i}} &\sim Poisson(X_{j,l}D_{l,i}G_i) \\ Z_{Z_{j,c,i}} &\sim Poisson(Z_{j,c}C_{c,i}G_i) \\ Y_{j,i} &= \sum_{l=1}^{r} Z_{X_{j,l,i}} + \sum_{c=1}^{k} Z_{Z_{j,c,i}} \end{aligned} \quad (2)$$

The EM iteration alternates between an E-step, which evaluates the expected likelihood over $Z_{X_{j,l,i}}$ and $Z_{Z_{j,c,i}}$ using the current set of parameters and a M-step, where the parameters are updated by maximizing the expected likelihood obtained in the E-step.

The parameter set $\theta = (X_{j,l}, Z_{j,c}, C_{c,i})$ is estimated in two stages: first, we estimate the expression centroids $X_{j,l}$ and $Z_{j,c}$ given $Y_{j,i}, D_{l,i}, C_{c,i}$ and G_i; second, the cluster memberships $C_{c,i}$ are updated given $Y_{j,i}, X_{j,l}, D_{l,i}$, $Z_{j,c}$ and G_i.

In the first stage, the parameter estimation algorithm is as described in Häkkinen *et al. (31)*. The full model likelihood from Eq (1) is given by:

$$logL(\boldsymbol{Y}) = \sum_{j=1}^{m}\sum_{i=1}^{n}\left(Y_{j,i}log\left(\sum_{l=1}^{r}X_{j,l}D_{l,i}G_i + \sum_{c=1}^{k}Z_{j,c}C_{c,i}G_i\right) - \left(\sum_{l=1}^{r}X_{j,l}D_{l,i}G_i + \sum_{c=1}^{k}Z_{j,c}C_{c,i}G_i\right)\right)$$

(3)

While the latent model likelihood is given by

$$logL(\boldsymbol{Z}) = \sum_{j=1}^{m}\sum_{i=1}^{n}\sum_{l=1}^{r} Z_{X_{j,l,i}} logX_{j,l}D_{l,i}G_i - X_{j,l}D_{l,i}G_i + \sum_{j=1}^{m}\sum_{i=1}^{n}\sum_{c=1}^{k} Z_{Z_{j,c,i}} logZ_{j,c}C_{c,i}G_i - Z_{j,c}C_{c,i}G_i \quad (4)$$

Let $\theta^0 = (X_{j,l}^0, Z_{j,c}^0, C_{c,i}^0)$ be the current set of parameters and

$$E_{Z_{X_{j,l,i}}} = E\left[Z_{X_{j,l,i}} | \theta^0, \sum_{l=1}^{r} Z_{X_{j,l,i}} + \sum_{c=1}^{k} Z_{Z_{j,c,i}} = Y_{j,i}\right]$$

$$E_{Z_{Z_{j,c,i}}} = E\left[Z_{Z_{j,c,i}} | \theta^0, \sum_{l=1}^{r} Z_{X_{j,l,i}} + \sum_{c=1}^{k} Z_{Z_{j,c,i}} = Y_{j,i}\right]$$

The derivatives of $logL(\boldsymbol{Z})$ with respect to $X_{j,l}$ and $Z_{j,c}$ are given by:

$$\frac{\partial logL(\boldsymbol{Z})}{\partial X_{j,l}} = \sum_{i=1}^{n} \frac{E_{Z_{X_{j,l,i}}}}{X_{j,l}} - D_{l,i}G_i$$

$$\frac{\partial logL(\boldsymbol{Z})}{\partial Z_{j,c}} = \sum_{i=1}^{n} \frac{E_{Z_{Z_{j,c,i}}}}{Z_{j,c}} - C_{c,i}G_i \quad (5)$$

and $logL(\boldsymbol{Z})$ is maximized at:

$$\hat{X}_{j,l} = \frac{\sum_{i=1}^{n} E_{Z_{X_{j,l,i}}}}{\sum_{i=1}^{n} D_{l,i}G_i}$$

$$\hat{Z}_{j,c} = \frac{\sum_{i=1}^{n} E_{Z_{Z_{j,c,i}}}}{\sum_{i=1}^{n} C_{c,i}G_i} \quad (6)$$

In the second stage, given $Y_{j,i}, D_{l,i}$, G_i and updated $X_{j,l}, Z_{j,c}$ in the first stage, we update $C_{c,i}$by:

$$\widehat{C_{c,i}} = \begin{cases} 1, \text{ iff c} = \arg\max_{c=1,2,\ldots,k} \sum_{i=1}^{n} E_{Z_{Z_{j,c,i}}} logZ_{j,c}C_{c,i}G_i - Z_{j,c}C_{c,i}G_i \\ 0, \text{ otherwise} \end{cases} \quad (7)$$

To obtain the latent mean of $Z_{Z_{j,c,i}}$ and $Z_{X_{j,c,i}}$ corresponding to the updated parameters, consider:

$$Z_{X_{j,l,i}} \sim Poisson(X_{j,l} D_{l,i} G_i)$$
$$Z_{Z_{j,c,i}} \sim Poisson(Z_{j,c} C_{c,i} G_i) \tag{8}$$
$$Z_{X_{j,l,i}} + Z_{Z_{j,c,i}} | \sum_{l=1}^{r} Z_{X_{j,l,i}} + \sum_{c=1}^{k} Z_{Z_{j,c,i}} = Y_{j,i} \sim \mathcal{B}\left(Y_{j,i}, \frac{Z_{X_{j,l,i}} + Z_{Z_{j,c,i}}}{\sum_{l=1}^{r} Z_{X_{j,l,i}} + \sum_{c=1}^{k} Z_{Z_{j,c,i}}} \right)$$

For $K \sim \mathcal{B}(y, p)$:

$$E[K|y] = py \tag{9}$$

Finally, we summarize the parameter estimation procedure as follows:

1. Choose initial values for the parameters $\theta^0 = (X_{j,l}^0, Z_{j,c}^0, C_{c,i}^0)$. We initialize $X_{j,l} = 1$, $Z_{j,c} = 1$ and $C_{c,i}$ uniform random.
2. Obtain the latent mean of $Z_{Z_{j,c,i}}$ and $Z_{X_{j,c,i}}$ using Eq. (8) and (9).
3. Update $X_{j,l}$, $Z_{j,c}$ using Eq. (6).
4. Obtain the latent mean of $Z_{Z_{j,c,i}}$ and $Z_{X_{j,c,i}}$ using Eq. (8) and (9).
5. Update $C_{c,i}$ using Eq. (7).
6. Repeat 2-5 until convergence.

Estimation of denoised expression profile for individual cells. Given $Y_{j,i}$, $D_{l,i}$, G_i and the estimated parameter set $\theta = (X_{j,l}, Z_{j,c}, C_{c,i})$, we computed $\tilde{Z}_{j,i}$, the denoised expression of gene j in the i^{th}cell. Considering:

$$Y_{j,i} \sim Poisson\left(\left(\sum_{l=1}^{r} (X_{j,l} D_{l,i}) + \tilde{Z}_{j,i} \right) G_i \right) \tag{10}$$

More generally, for a given subset of cells I, the full model likelihood is analogous to Eq (3), and the derivatives with respect $\tilde{Z}_{j,I}$ are:

$$\frac{\partial log L(\boldsymbol{Y})}{\partial \tilde{Z}_{j,I}} = \sum_{i \in I} \left(\frac{Y_{j,i} G_i}{\sum_{l=1}^{r} X_{j,l} D_{l,i} G_i + \tilde{Z}_{j,I} G_i} - G_i \right) \tag{11}$$

when $I = \{ i \}$ contains a single cell, the root is:

$$\tilde{Z}_{j,i} = \frac{\left(Y_{j,i} - \sum_{l=1}^{r} X_{j,l} D_{l,i} G_i \right)}{G_i}, \tilde{Z}_{j,i} \geq 0 \tag{12}$$

and for a general subset of cells $\tilde{Z}_{j,I}$ can be found by bisecting Eq (11).

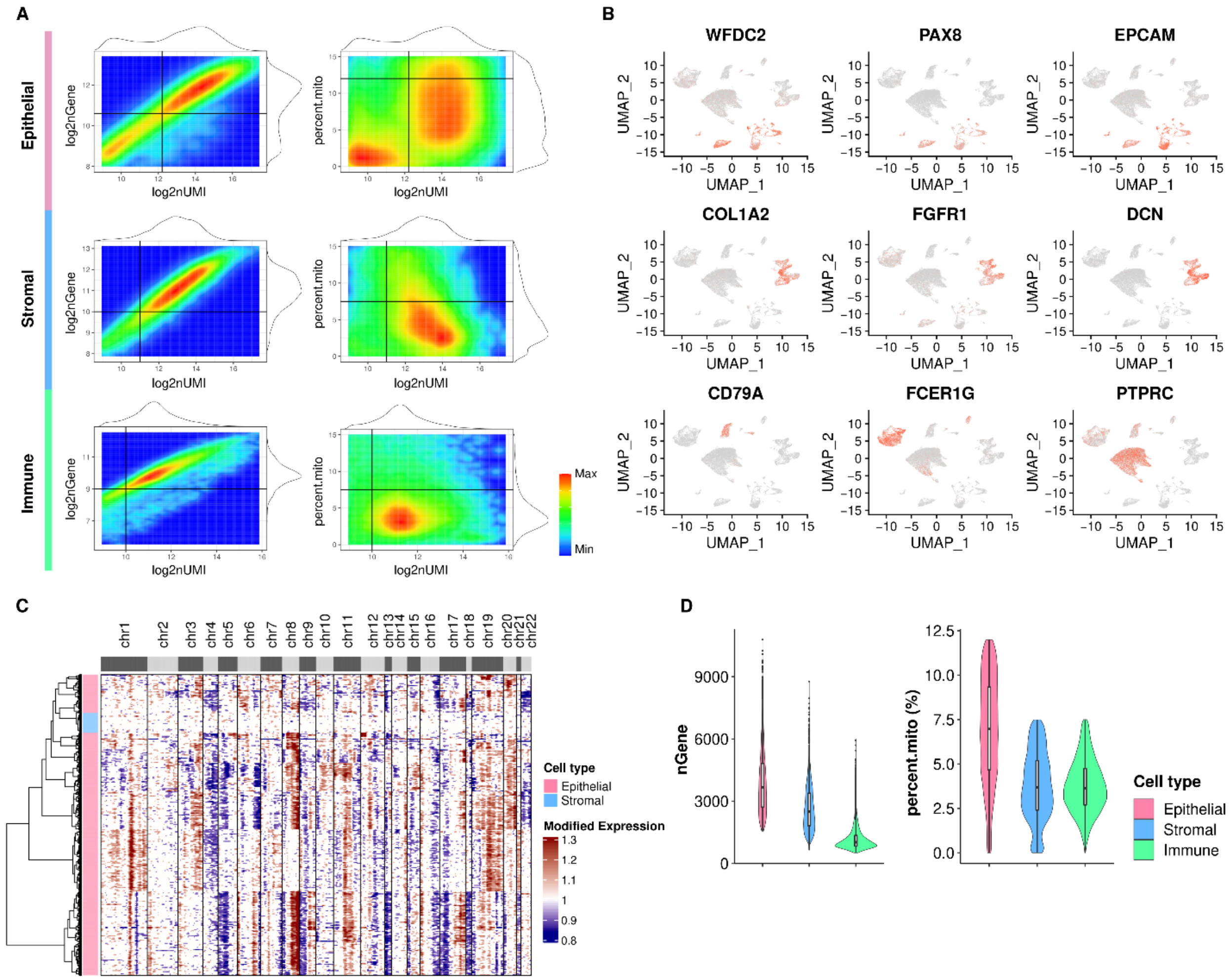

Fig. S1. Quality control of sc-RNAseq data and identification of major cell types. (**A**) Density plots showing the quality measures (log2nUMI, log2nGene, percent of mitochondrial derived transcripts (percent.mito)) and the corresponding cutoffs in epithelial, stromal and immune cells. (**B**) UMAP plots showing the expression of acknowledged markers of tumor, stromal and immune cells after quality control. Cells (dots) are colored by the expression level of each marker. Red indicates a higher level while grey indicates a lower level of expression. (**C**) Inferred CNA profiles of epithelial, and stromal cells after quality control. (**D**) Violin plot of number of the genes (nGene) and percent of mitochondrial derived transcripts (percent.mito) in epithelial, stromal and immune cells after quality control.

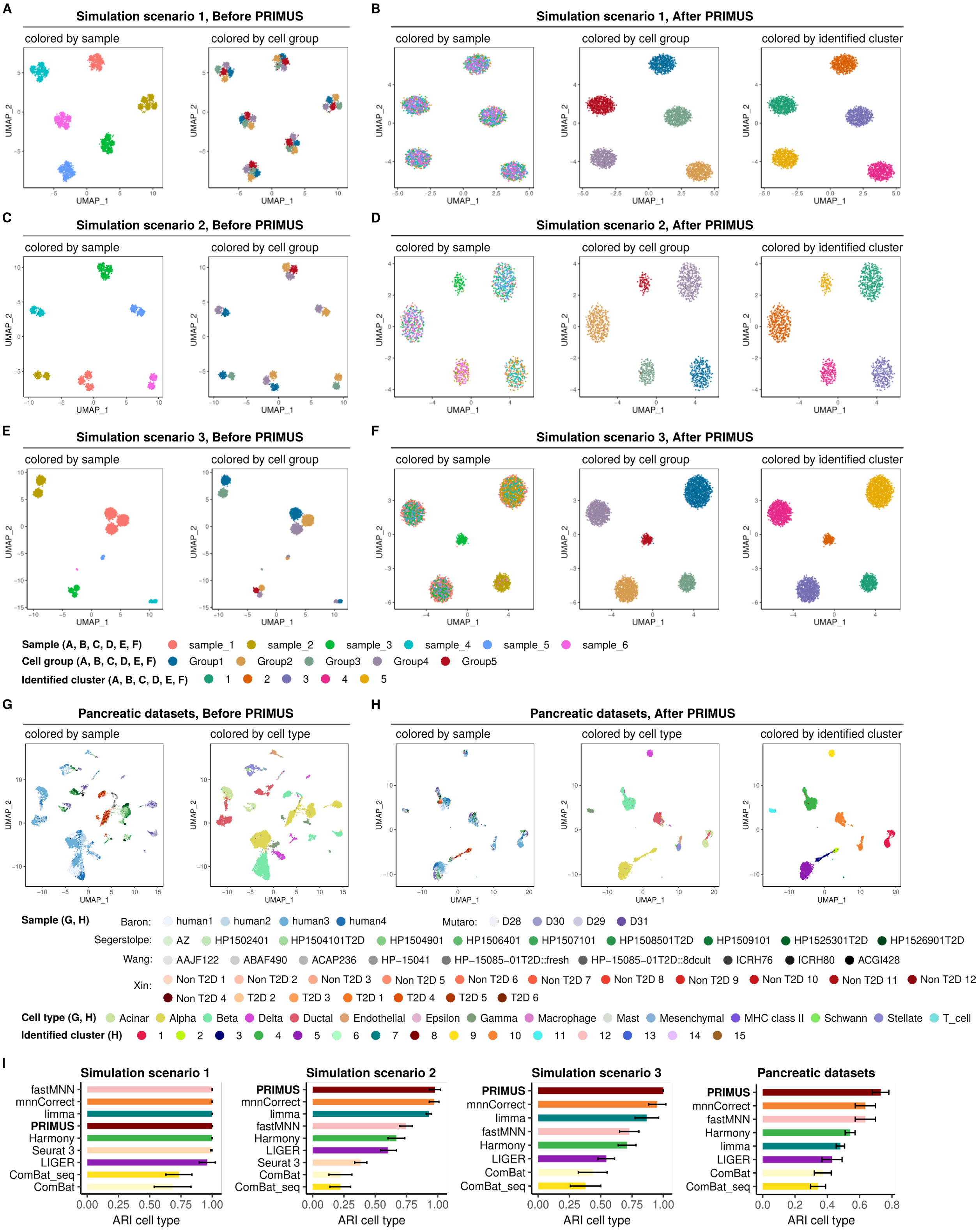

Fig. S2. Benchmarking integration of phenotypic groups across multiple samples and data sets. (**A-H**) UMAP embeddings of example datasets in simulation scenario 1 (**A, B**), simulation

scenario 2 (**C, D**), simulation scenario 3 (**E, F**) and pancreatic datasets (**G, H**), before and after PRIMUS integration, colored by sample, cell group/type and PRIMUS identified clusters. (**I**) Barplots showing ARIs between cell groups/types and the identified clusters using PRIMUS and eight comparison methods (fastMNN, mnnCorrect, limma, Harmony, Seurat v3, LIEGR, ComBat-seq and ComBat) for the simulated datasets (20 datasets in each simulation scenario) and the pancreatic datasets (randomly sampled 80% of the cells 20 times).

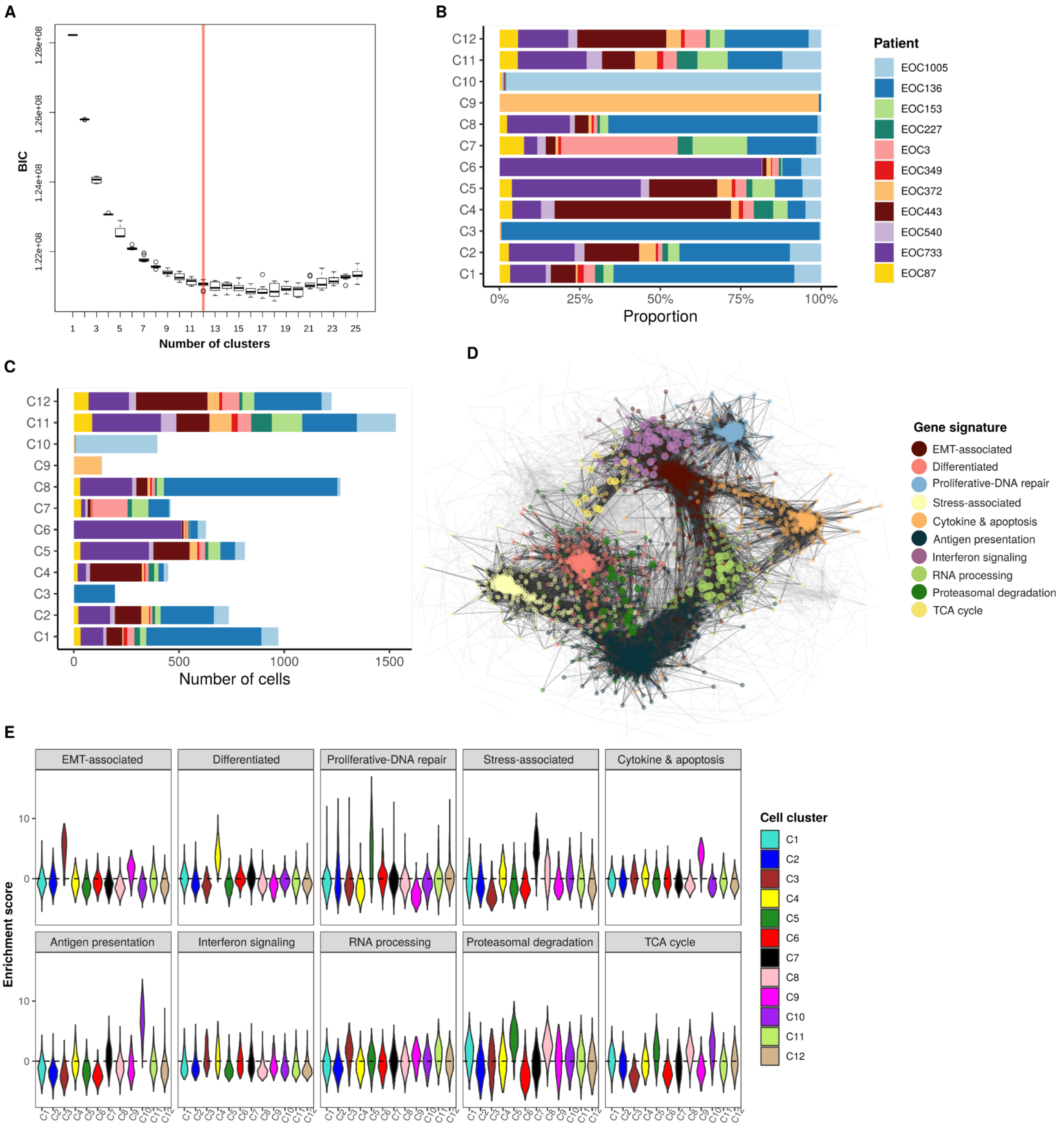

Fig. S3. Model selection for cancer cell clustering and characterization of cancer cell clusters. (**A**) Selection of the number of cancer cell clusters based on Bayesian information criterion (BIC). The boxplot showing the BIC of models fitted for $k = 1, 2, \ldots, 25$ with 10 different random initial parameter sets for each k. The red line indicates the selected number of clusters ($k = 12$). **(B)** Barplot showing the proportion of cells from each patient in each cluster. Each bar corresponds to one cancer cell cluster, colored based on patients. **(C)** Barplot showing the number of cells from each patient in each cluster. Each bar corresponds to one cancer cell cluster, colored based on patients. (**D**) Force-directed embedding layout of the network with the 4742 differentially expressed genes (DEGs) as vertices and their Pearson correlations of the likelihood-ratio test

statistic as edges. Colors denote the gene communities to which the cells are assigned, and the unassigned genes are omitted. (**E**) Violin plots showing enrichment scores of each gene signature for individual cells in each cancer cell cluster. Colors denote cancer cell clusters.

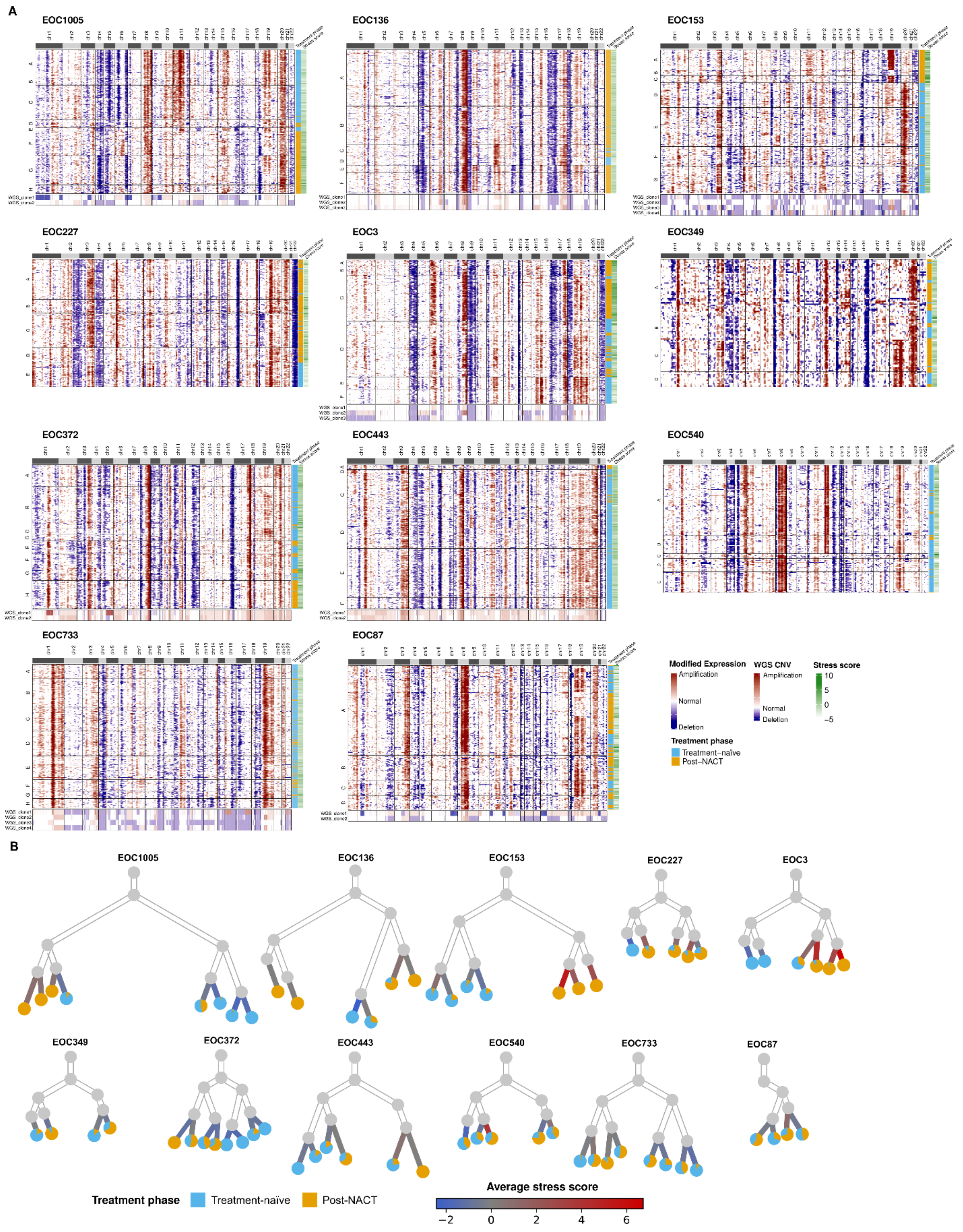

Fig. S4. CNA profiles and subclonal structures inferred from scRNA-seq data. (**A**) Heatmaps showing the inferred subclonal CNA profiles of the 11 patients with scRNA-seq data. The

annotation bar below each heatmap shows the subclonal CNA profiles from the bulk whole-genome sequencing data from the same patients. (**B**) Clonality trees of each of the 11 patients, inferred from the scRNA-seq data. Each leaf node represents a CNA subclone, shown as a pie chart displaying the relative proportion of treatment-naïve (blue) and post-NACT (yellow) cells. The branches leading to the leaf nodes are colored by stress scores of the corresponding subclones.

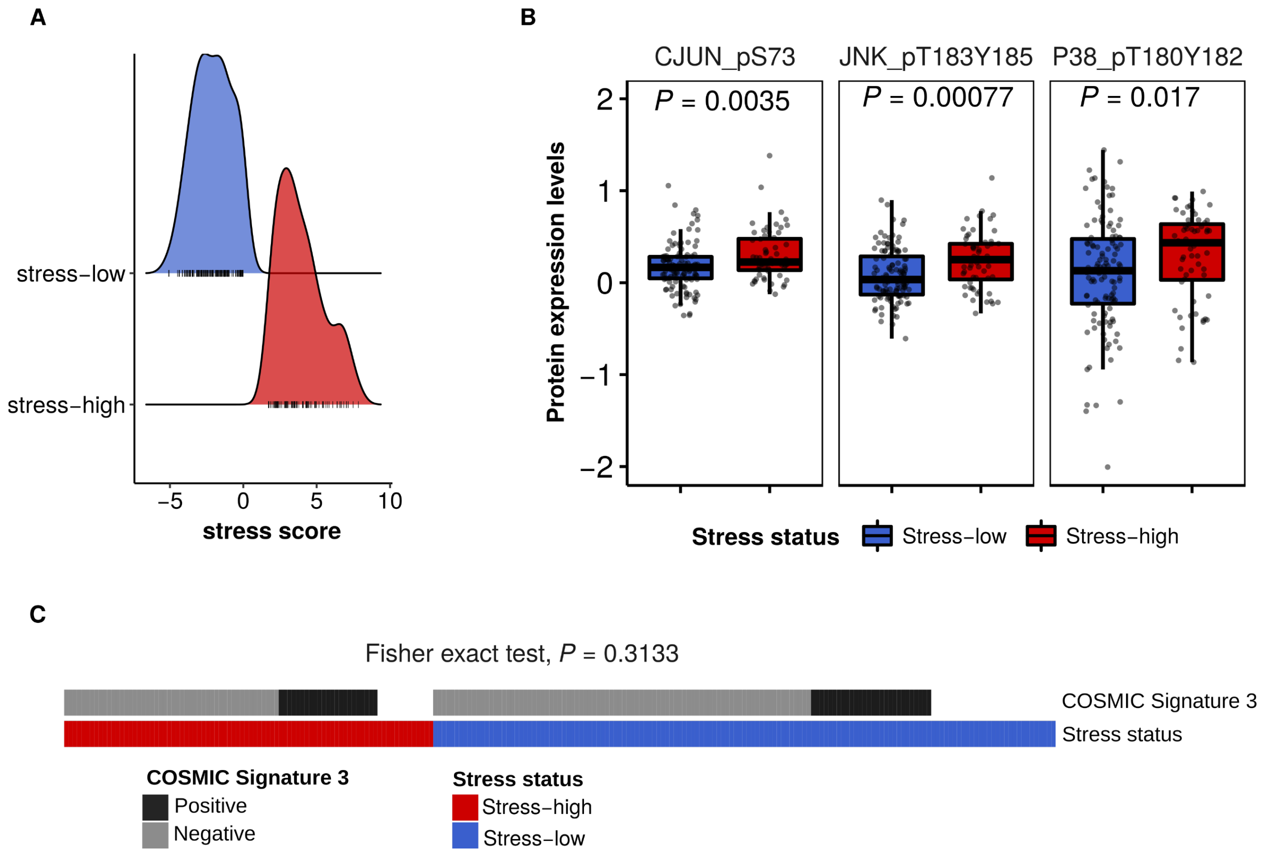

Fig. S5. TCGA reverse phase protein array data confirmed the stress state on protein level. (**A**) Density plots showing the stress scores of stress-high and stress-low samples in the TCGA cohort. (**B**) Box plots comparing the protein expression levels of c-JUN_pS73 (P = 0.0035), JNK_pT183Y185 (P = 0.00077), P38_pT180Y182 (P = 0.017) between stress-high and stress-low patients from the TCGA cohort. P values were computed using the Wilcoxon rank-sum test. (**C**) COSMIC signature 3 status is not enriched in stress-high or stress-low patients in the TCGA cohort (Fisher exact test, P = 0.3133).

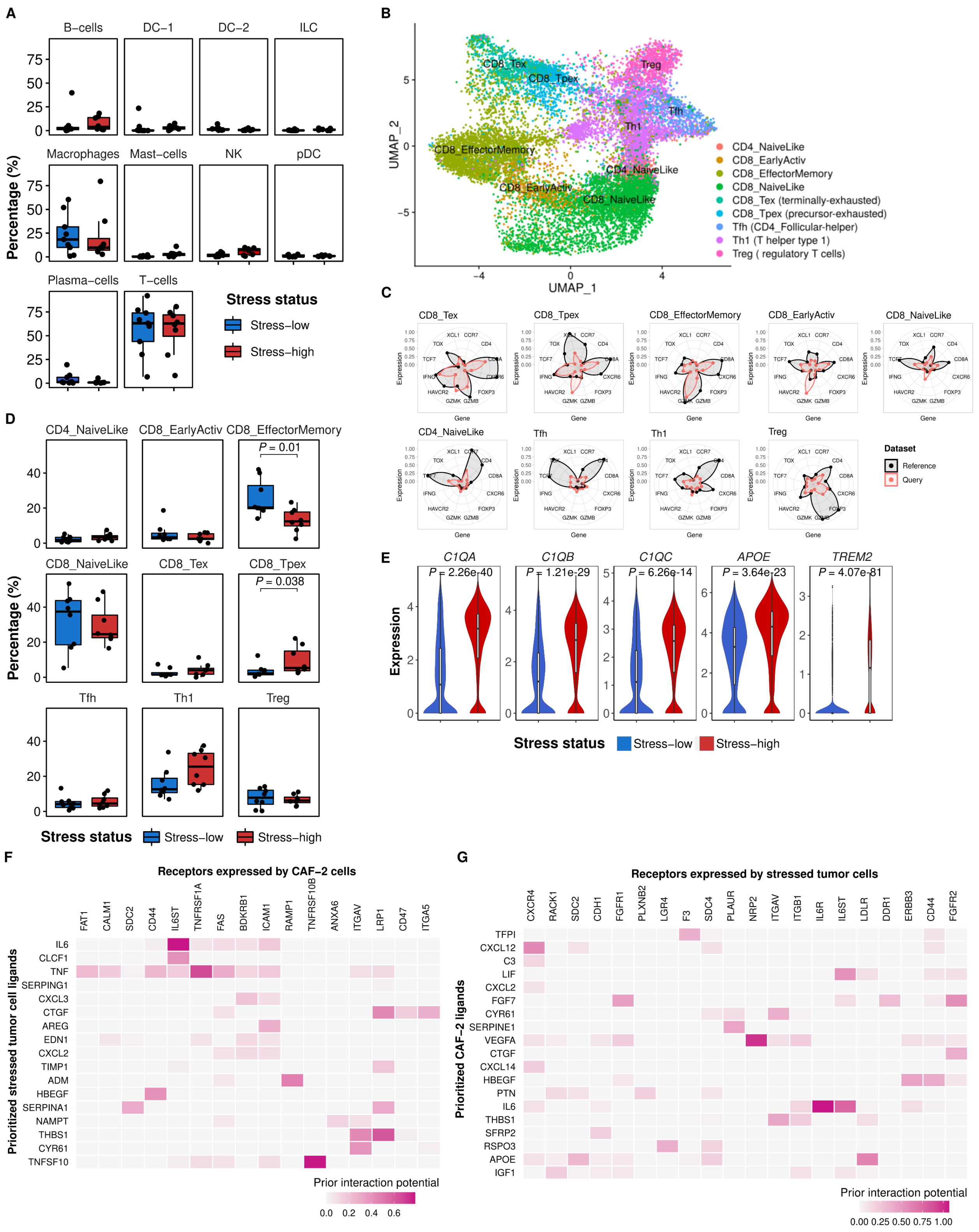

Fig. S6. Immune tumor microenvironment and potential receptors for stromal interactions in stress-high tumors. (**A**) Boxplots showing the fractional differences (Wilcoxon rank-sum test) of identified immune subtypes between stress-high (red) and stress-low tumors (blue). Each dot

represents a tumor sample. All differences with FDR adjusted $P < 0.05$ are indicated. (**B**) UMAP showing the identified T-cell subtypes by projecting the T-cells to a reference atlas using ProjecTILs, colored by cell type. (**C**) Normalized expression of T-cell subtype marker genes in T-cells in our cohort (red) and the reference cells (black). (**D**) Boxplots showing the fractional differences (Wilcoxon rank-sum test) of identified T-cell subtypes between stress-high (red) and stress-low tumors (blue). Each dot represents a tumor sample. All differences with $P < 0.05$ are indicated. (**E**) Violin plots showing the expression of immunosuppressive phenotype marker genes (*C1QA, C1QB, C1QC*, *APOE*, *TREM2*) in macrophages in stress-high (red) and stress-low (blue) samples. (**F**) Heatmap showing the interactions of the prioritized ligand in stressed cancer cells with the receptors expressed in the inflammatory stroma (CAF-2). (**G**) Heatmap showing the interactions of the prioritized ligand in the inflammatory stroma (CAF-2) with the receptors expressed in stressed cancer cells.

Simulation scenario	Sample	Group1	Group2	Group3	Group4	Group5	Total
Scenario 1	sample_1	100	100	100	100	100	500
	sample_2	100	100	100	100	100	500
	sample_3	100	100	100	100	100	500
	sample_4	100	100	100	100	100	500
	sample_5	100	100	100	100	100	500
	sample_6	100	100	100	100	100	500
Scenario 2	**Sample**	**Group1**	**Group2**	**Group3**	**Group4**	**Group5**	**Total**
	sample_1	100	100	-	100	-	300
	sample_2	100	-	100	-	-	200
	sample_3	-	100	-	100	100	300
	sample_4	100	-	-	100	-	200
	sample_5	-	100	-	100	-	200
	sample_6	-	100	100	-	-	200
Scenario 3	**Sample**	**Group1**	**Group2**	**Group3**	**Group4**	**Group5**	**Total**
	sample_1	640-699	638-694	-	643-690	-	2000
	sample_2	481-519	-	481-519	-	-	1000
	sample_3	-	152-187	-	141-183	150-177	500
	sample_4	84-116	-	-	84-116	-	200
	sample_5	-	43-59	-	41-57	-	100
	sample_6	-	7-13	7-13	-	-	20

Table S1. Number of cells in each group from each sample in three simulation scenarios.

Gene signature	Genes
EMT-associated (43 genes)	*DST, EGFR, EPHB2, ITGA6, PIK3CD, PLEC, SFN, SMAD3, CLTC, EIF2AK2, ITGA2, KPNA1, STAT1, BCAR1, CCND1, COL1A2, ITCH, MMP1, MMP12, PML, TNC, CDK6, FADD, FAS, MX1, E2F3, BIRC2, CAPN2, VASP, MMP9, DFFA, KPNB1, ADAMTS5, MMP10, AP2B1, ASAP1, SDCBP, CYP1A1, CYP1B1, CYP3A5, MAFG, SLC7A11, IGF2*
Differentiated (40 genes)	*B3GNT7, B3GNT8, GCNT3, MUC15, MUC4, MUC5B, ST3GAL4, LCN2, S100A8, S100A9, ALOX5, AOC1, B2M, C3, CEACAM1, CEACAM6, MGST1, OSTF1, PPBP, S100P, SLPI, TMEM173, DSC2, EVPL, IVL, KRT13, KRT4, KRT6A, KRT6C, HRASLS2, LPCAT4, PLBD1, RARRES3, CDKN2B, STEAP4, TGFB1, CFB, CCL28, CX3CL1, CXCL10*
Proliferative-DNA repair (106 genes)	*AURKA, AURKB, BIRC5, BUB1, BUB1B, CCNA2, CCNB1, CCNB2, CDC20, CDC25C, CDC45, CDC6, CDCA8, CDK1, CDT1, CENPA, CENPE, CENPF, CENPH, CENPK, CENPM, CENPN, CENPU, CKAP5, DHFR, E2F1, H2AFV, H2AFZ, HAUS1, HIST1H2BH, HIST1H3G, HIST1H4C, HIST2H2AC, KIF18A, KIF20A, KIF23, KIF2C, LIG1, LMNB1, MAD2L1, MCM10, MCM2, MCM3, MCM4, MCM5, MCM6, MCM7, MCM8, NDC80, NEK2, NUF2, ORC6, PCNA, PLK1, PLK4, POLD1, POLD2, PRIM1, PTTG1, RFC2, RFC3, RFC4, RRM2, SGO1, SGO2, SKA1, SKA2, SKP2, SPC24, SPC25, TK1, TMPO, TPX2, UBE2C, VRK1, ZWILCH, ZWINT, BRCA1, CHEK1, CLSPN, RHNO1, RMI2, FANCG, HMGB2, KIF4A, RRM1, TYMS, RACGAP1, CBX5, BRCA2, FANCD2, RAD51, RAD51AP1, FANCB, FANCI, UBE2T, KIF11, KIF15, KIF20B, KIF22, KIFC1, HIST1H1A, HIST1H1B, GGH, DTYMK, AKR1B1*
Stress-associated (35 genes)	*CEBPB, CEBPD, FOS, IL6, JUN, JUNB, MCL1, MYC, SOCS3, ATF3, DUSP1, EGR1, FOSB, CEBPA, DDIT3, EGR2, CDKN1A, GADD45B, TNF, HES1, HBEGF, BCL6, NR4A1, DUSP6, GADD45G, ID2, NFKBIA, PLK3, SNAI2, CREB5, HLA-G, HIST1H2BC, HIST1H2BG, CALML3, SNAI1*
Cytokine & apoptosis (11 genes)	*CCL20, CXCL1, IL1R2, IL1RN, SEC11C, SPCS3, BIK, BIRC3, CDKN2A, DAB2, GJB2*
Antigen presentation (82 genes)	*CTSL, HLA-DMA, HLA-DMB, HLA-DOA, HLA-DPA1, HLA-DPB1, HLA-DQA1, HLA-DQA2, HLA-DRA, HLA-DRB5, HSP90AB1, HSPA2, HSPA4, HSPA5, HSPA6, PDIA3, CPE, CTSF, C1R, CXADR, SFTPD, TUBB2A, TUBB6, FOLR1, GAS6, NPC2, RUNX1, TGFBR2, GNAI1, RBPJ, IL11RA,*

	HIST1H2AE, DNAJC3, FBXO2, HERPUD1, HSP90B1, SKP1, UBE2D1, UBQLN1, NECTIN2, SDC2, CCND3, DNAJB9, ERP27, SIAH1, PPP2CA, BAG3, HSPA12A, BMP2, LATS2, PPP2R2B, PRKCI, WNT6, IFITM1, IFITM2, CRMP1, DPYSL3, PLXNA2, MYL9, TNNC1, TNNI1, TNNT2, TLE1, TLE4, APP, PELI2, VAMP2, DNAJA4, PLAT, ACADVL, TSPYL2, NR1D2, PURA, GATA6, TWIST1, RING1, BAMBI, ATG101, RAPGEF2, NR4A3, SOX4, SLC22A18
Interferon signaling (11 genes)	*GBP4, IFI27, IFI35, IFIT1, ISG15, OAS1, OAS2, STAT2, TRIM22, TAP1, ITGAV*
RNA processing (20 genes)	*BMS1, DCAF13, MPHOSPH10, PNO1, ACIN1, ROCK1, TJP1, ESCO1, WAPL, EIF1AX, PNN, RNPS1, DYNC1LI1, BDP1, CRCP, PAFAH1B1, PRPF40A, SFSWAP, KMT2A, SETD2*
Proteasomal degradation (39 genes)	*AIMP1, EIF3I, MRPL13, MRPL15, MRPL16, MRPL2, MRPL28, MRPL3, MRPS7, RPN2, SRP9, TSFM, PSMA4, PSMB5, PSMB6, PSMB7, PSMC5, PSMD8, ACTL6A, BANF1, PPIA, XRCC6, VDAC3, FH, MDH2, SDHB, TPI1, ECHS1, HSD17B10, POLR2G, NDUFB6, HNRNPA2B1, HNRNPA3, SNRPD3, PRDX3, PTMA, CCT7, VBP1, COPE*
TCA cycle (20 genes)	*GLO1, IDH3B, NDUFA10, NDUFB5, PDHA1, SUCLG1, TRAP1, UQCRC1, UQCRC2, MDH1, ALDH7A1, DECR1, ECH1, ECI2, PCCB, SHMT1, HACD3, PTGR1, MGST2, PRDX6*

Table S2. List of genes in each gene signature.

Probe name	Catalogue number
Hs-MYC	#311761
Hs-TNF-a	#310421
Hs-DDIT	#311131
Hs-NR4A1	#851021
Hs-ATF3-C2	#470861-C2
Hs-HBEGF-C2	#524821-C2
Hs-FOS-C2	#319901-C2
Hs-JUN-C3	#470541-C3
Hs-CEBPD-C3	#831431-C3
Hs-CEBPB-C3	#831431-C3
Hs-3-plex positive control probe, POLR2A, PPIB, UBC	#320861
3-Plex negative control probe DapB	#320871

Table S3. RNAscope probes used for 10 stress signature genes and controls.

Set	Probe	Fluorophore
Set 1	MYC	FITC
	ATF3-C2	TRITC
	CEBPD-C3	Cy5
Set 2	NR4A1	Cy5
	FOS-C2	FITC
	JUN-C3	TRITC
Set 3	DDIT	FITC
	ATF3-C2	TRITC
	CEBPD-C3	Cy5
Set 4	TNFA	Cy5
	HBEGF-C2	TRITC
	CEBPB-C3	FITC

Table S4. Probe combinations and fluorophore channels used for 10 stress signature genes.

Gene	Coefficient
ATF3	0.0867
CEBPB	-0.1527
CEBPD	-0.1468
DDIT3	0.0018
FOS	0.0735
HBEGF	0.2109
JUN	0.1430
MYC	-0.0555
NR4A1	0.4647
TNF	0.1025

Table S5. The coefficients for each gene in the first canonical component of the RNA-ISH data.

Data S1.

List of differentially expressed (DE) genes higher expressed in CAF-2 versus CAF-1 and CAF-3.

Data S2.

List of differentially expressed (DE) genes higher expressed in stressed cancer cells versus other cancer cells.
